# Quantum Theoretical, Chemo-Computational, and Comparative Molecular Coupling Studies of Two Enantiomeric Naphthoquinones, Alkannin and Shikonin, and Their Corresponding Indole Derivatives

**DOI:** 10.3390/molecules31183275

**Published:** 2026-09-16

**Authors:** Adriana Lizbeth Rivera Espejel, Sebastian Buendia Lira, René Miranda Ruvalcaba, René Gerardo Escobedo-González, Miguel Cuevas-Cruz, Alberto A. Fajardo de la Rosa, David A. Nieto-Álvarez, Joel Martínez, María Inés Nicolás-Vázquez

**Affiliations:** 1 Facultad de Estudios Superiores Cuautitlán, Departamento de Ciencias Químicas, Campo 1, Universidad Nacional Autónoma de México, Cuautitlán Izcalli 54740, Mexico; adriana.rivera@cuautitlan.unam.mx (A.L.R.E.); buendiasebastian15@gmail.com (S.B.L.); mirruv@comunidad.unam.mx (R.M.R.); 2 Departamento de Mantenimiento Industrial y Nanotecnología, Universidad Tecnológica de Juárez, Ciudad Juárez 32695, Mexico; renegerardo.escobedo@gmail.com; 3 Departamento de Oceanografía Biológica, Centro de Investigación Científica y de Educación Superior de Ensenada (CICESE), Ensenada 22860, Mexico; mcuevas@cicese.edu.mx; 4 Laboratorio de Ingeniería Química, Facultad de Química, Universidad Nacional Autónoma de México, Alcaldía Coyoacán, Ciudad de México 04510, Mexico; aafdlr@ciencias.unam.mx; 5 Instituto Mexicano del Petróleo, Eje Central Lázaro Cárdenas Norte 152, San Bartolo Atepehuacan, Ciudad de México 07730, Mexico; dnieto@imp.mx

**Keywords:** alkannin, shikonin, indole derivatives, in silico, DFT, molecular docking, COX-2, PARP-1, HDAC2

## Abstract

The use of computational tools has become widespread in drug discovery as an effective approach to guide the development of new therapeutic agents, including potential cancer drugs. In addition, plant-derived secondary metabolites have attracted considerable interest as a valuable source of bioactive compounds. Among these, alkannin and shikonin, two enantiomeric phyto naphthoquinones, have emerged as promising candidates for cancer therapy because of their anti-inflammatory properties and their ability to induce oxidative DNA damage in malignant cells. Related to the above commentaries, in the present study, the phyto naphthoquinones alkannin, shikonin, and their corresponding indole derivatives were evaluated employing density functional theory (DFT), followed by chemoinformatic analyses and molecular docking studies to evaluate, in silico, their potential activity against the cancer-related enzymes PARP-1, COX-2, and HDAC2, which are overexpressed in several types of cancer. The presence of the indole moiety in the phytomolecules increased their chemical reactivity, as exhibited by the calculated properties and the smaller LUMO–HOMO energy gap. In addition, the bioinformatic evaluation predicted that the target molecules may act as potential apoptosis agonists and antineoplastic agents. Finally, the performed molecular docking analysis suggested a stereoselective effect on the stability of ligand–protein binding complexes, particularly for alkannin, including relevant interactions with His201 and its indole derivative displaying interactions with His201, Tyr235, and Glu327 from the PARP-1 catalytic triad. Additionally, alkannin (−11.2 kcal/mol) and the corresponding indole derivative of shikonin (−10.14 kcal/mol) showed better interaction energies with COX-2 than naproxen (−8.75 kcal/mol). In the latter case, the inclusion of the indole moiety improved the binding affinity. In contrast, the affinity of the target compounds for HDAC2 decreased considerably with the indole derivatives in comparison with the reference compound BIZ.

## 1. Introduction

The search for anticancer agents derived from secondary metabolites has attracted considerable attention, and phytochemistry has emerged as an important source of novel antineoplastic compounds. Among these natural products, quinones and their derivatives have received particular interest because of their broad spectrum of biological activities [1,2,3].

Currently, breast cancer affects approximately 1 in 20 women worldwide, and its incidence is expected to reach 3.2 million new cases, with approximately 1.1 million deaths by 2050 [4]. Hence, it is convenient to note that given the urgent need for new therapeutic options, several studies have documented some phytoquinones and their indole derivatives as promising antineoplastic candidates because of their affinity for poly(ADP-ribose) polymerase-1 (PARP-1), an enzyme that is overexpressed during neoplastic processes [5,6].

**Figure 1:**
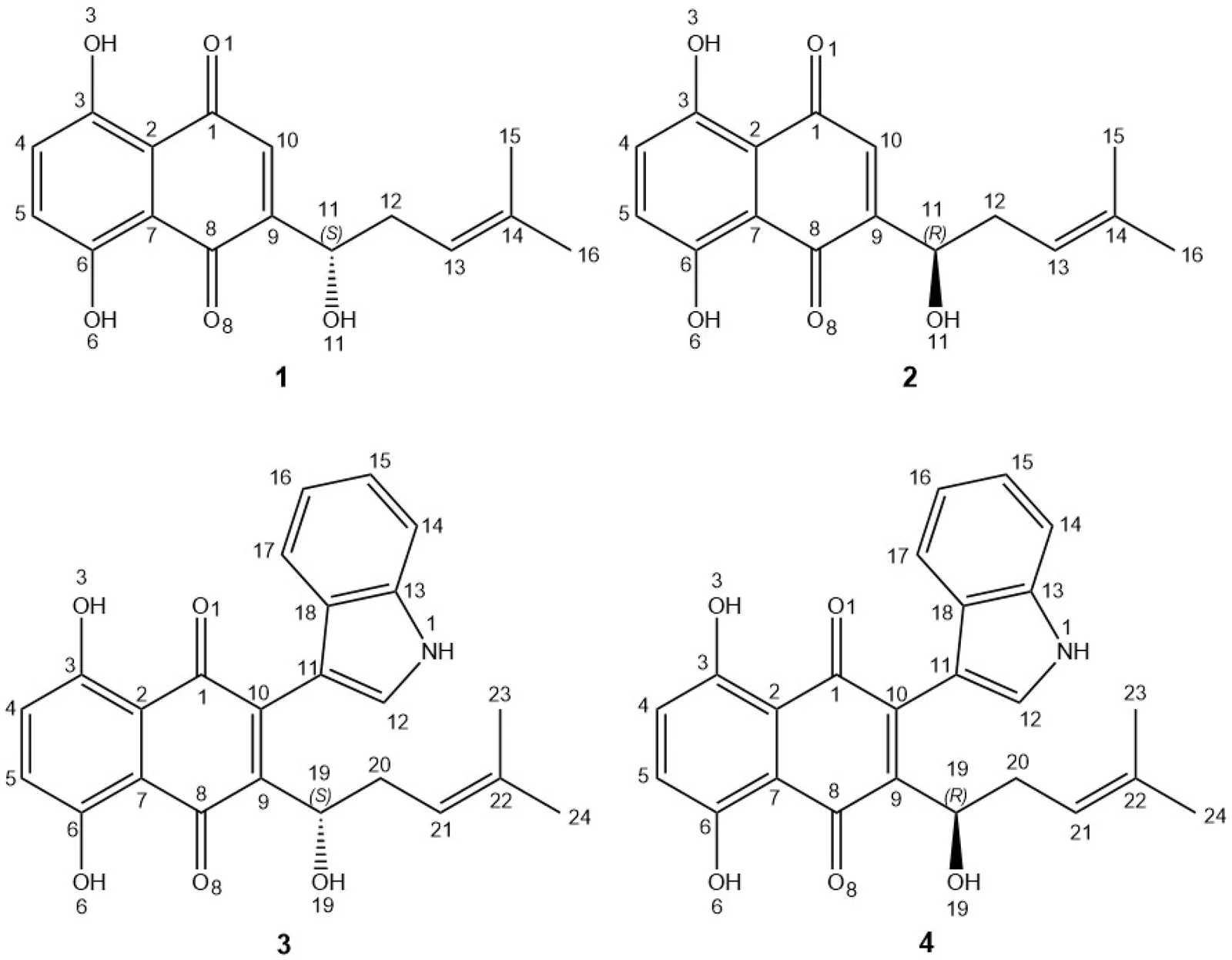
Structures and proposed enumeration for alkannin (**1**), shikonin (**2**), and indolyl naphthoquinones (**3**,**4**).

Following previous studies on plant-derived quinones, particular attention has been focused on two naturally occurring enantiomeric naphthoquinones (Figure 1): alkannin (**1**), isolated from the roots of *Alkanna tinctoria*, a plant native to southern and central Europe, the Mediterranean region, and Turkey; shikonin (**2**), isolated from *Lithospermum erythrorhizon*, a species native to China, Japan, and Korea [7].

The interest in studying the enantiomeric pair alkannin/shikonin lies in the unusual occurrence of each enantiomer in a different plant species. This feature is particularly relevant because both enantiomers may exhibit distinct pharmacological activities arising from their different stereochemical properties. Furthermore, the synthesis of two new indole derivatives (**3**, **4**), as shown in Figure 1, may further improve or enhance their pharmacological potential.

Previous studies have reported that **1** exhibits activity against colorectal cancer cells by inducing oxidative DNA damage selectively in malignant cells while sparing normal cells. Moreover, a synergistic antitumor effect has been proposed when alkannin is combined with olaparib [8]. Compound **2**, in turn, has demonstrated antibacterial, anti-inflammatory, and anticancer activities against breast, gastric, lung, hepatocellular, and thyroid carcinoma, as well as melanoma and glioblastoma [9]. In this sense, compounds **1**–**4** were evaluated as a possible potential inhibitors of histone deacetylase 2 (HDAC2) and cyclooxygenase-2 (COX-2), enzymes that are frequently overexpressed in several types of cancer, including lung, colon, breast, and bone tumors, where their inhibition has been associated with antitumor and pro-apoptotic effects [10,11].

Likewise, numerous indole derivatives have exhibited promising anticancer properties, particularly the diindolylmethanes, some of which are currently marketed as dietary supplements [12,13,14]. It is also noteworthy that the indolequinone moiety is found in *Aspergillus* sp. [15], which exhibits a wide range of biological activities, including insulin-mimetic, antiviral, and cytotoxic effects against cancer cells [16,17,18,19,20]. Consequently, indolyl quinones have attracted considerable interest as scaffolds for developing new biologically active molecules. Nevertheless, before undertaking their synthesis, it is essential to evaluate whether newly designed compounds are likely to possess favorable pharmacological properties [15]. In this context, computational methods have become indispensable tools for the rational design of new drug candidates [21].

Computational approaches enable the prediction of potential drug candidates before their synthesis [22]. These strategies have become an integral part of modern pharmaceutical research, facilitating the optimization of known bioactive molecules, reducing undesirable side effects, and promoting the development of compounds with improved selectivity and efficacy [23,24]. Consequently, computational methodologies have played an increasingly important role in drug discovery by supporting the identification, optimization, and validation of pharmacologically relevant molecules [25]. The term in silico refers to computational modeling, simulation, and visualization of biological and medical processes. Advances in medical informatics and computer technologies, particularly following the mapping of the human genome [26], have greatly strengthened the identification and evaluation of molecules with potential pharmacological activity [25]. Therefore, a comprehensive computational investigation integrating density functional theory (DFT), chemoinformatic analyses, and molecular docking studies is of considerable interest for expanding the current knowledge of naphthoquinone-based compounds.

As part of our ongoing research related to the pharmacological properties of molecules of the class of naphthoquinones in addition to various indole derivatives [17,20,27,28,29,30,31,32,33], and our aim to highlight our recent theoretical and chemoinformatic study of four indolyl naphthoquinones as potential anticancer candidates [6], the present work offers a comprehensive theoretical, computational, chemoinformatic, and molecular docking study of the enantiomeric phytoproducts alkannin and shikonin, and their corresponding indole derivatives (**1**–**4**). We evaluate their physicochemical and pharmacological properties, predict their potential antineoplastic activity, and identify promising candidates for future synthesis and experimental biological evaluations.

## 2. Results and Discussion

### 2.1. Ligand Optimization

**Figure 2:**
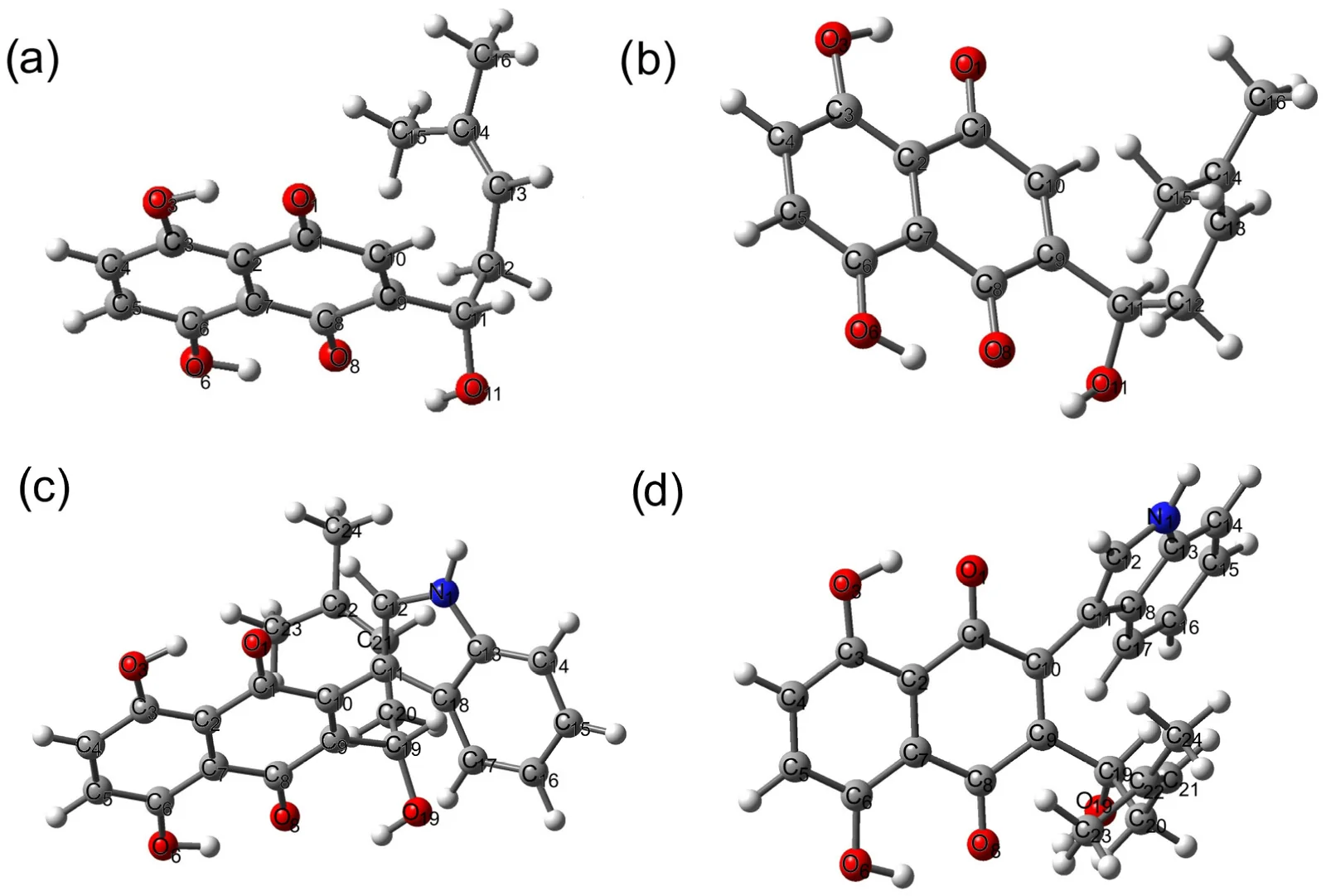
Theoretically optimized representations of (**a**) **1**, (**b**) **2**, (**c**) **3**, and (**d**) **4** most stable conformers.

The initial step in evaluating the target molecules was to analyze their geometries; a conformational analysis was first performed. Subsequently, the lowest-energy conformer was selected for each molecule and fully optimized using DFT with the B3LYP functional and the 6-311++G(d,p) basis set. The optimized molecular structures are shown in Figure 2.

The molecular geometries of the four compounds were optimized using density functional theory. The calculated electronic energies were −624,790.583 kcal/mol for alkannin (**1**), −624,790.583 kcal/mol for shikonin (**2**), −852,379.193 kcal/mol for indolylalkannin (**3**), and −852,379.193 kcal/mol for indolylshikonin (**4**), with identical energies for both enantiomeric pairs.

### 2.2. Geometrical Parameters

It is worth noting that no experimental X-ray crystallographic data is available for the target molecules. Therefore, comparisons with structurally related compounds were performed to assess the reliability of the calculated geometries. The experimental structural data for plumbagin [34], the side chain of perezone [35], and the structurally related indolyl derivative 2-ethyl-5-(3-indolyl)oxazole [36] were compared with the calculated geometrical parameters of the studied molecules (Tables S1–S3, Supplementary Materials).

Regarding the naphthoquinone ring, the geometrical parameters of compounds **1**–**4** were compared with those of plumbagin [36]. The calculated bond lengths showed good agreement with the corresponding experimental values (Table S1). Likewise, the hydrocarbon side chain was compared with the corresponding side chain of perezone, revealing very similar bond lengths in both structures. Overall, the naphthoquinones and their corresponding indole derivatives exhibited highly similar bond lengths. The calculated carbonyl bond lengths ranged from 1.243 to 1.249 Å for compounds **1**–**2** and from 1.242 to 1.255 Å for compounds **3**–**4**, with the C1–O1 bond being the shortest carbonyl bond in all compounds. In compounds **3**–**4**, the N1–C12 and N1–C13 bond lengths ranged from 1.366 to 1.384 Å, whereas the N1–H bond length was approximately 1.006 Å. Furthermore, the hydroxyl O–H bonds were the shortest bonds in all compounds, with bond lengths ranging from 0.967 to 0.990 Å.

In addition, the calculated bond angles for compounds **1**–**4** are presented in Table S2 (Supplementary Materials). The results revealed that both the naphthoquinones and their corresponding indole derivatives exhibited highly similar bond angles, highlighting the structural similarities of their common molecular framework. Bond angles involving carbonyl groups, such as C10-C1-O1, O1-C1-C2, and C2-C3-O3, ranged from 119.8° to 122.8°. Also, the terminal side chain containing the double bond displayed bond angles ranging from 120.7° to 122.7° in all compounds. Related to the indole derivatives, bond angles associated with the nitrogen-containing ring ranged from 108.5° to 110.2°.

Finally, a dihedral angle (Table S3) is defined as the angle between two planes sharing a common bond and is commonly used to describe the spatial orientation of atoms around a bond. For double bonds, the preferred dihedral angles are typically close to 0° and 180° (*trans*), whereas single bonds generally allow greater rotational freedom [37]. In the present study, several dihedral angles were found to be close to 0° or 180°, reflecting the overall planarity of the molecular structures. For example, the C1–C2–C3–C4 dihedral angle was calculated to be −179.8°, 179.2°, −179.2°, and 179.3° for compounds **1**–**4**, respectively. Similarly, the C13–C14–C15–C16 dihedral angle in the indole moiety of compounds **3** and **4** was 0.150° and −0.090°, respectively. In contrast, the terminal side chain exhibited the largest variations in dihedral angles, indicating that this region possesses the greatest conformational flexibility due to rotation about single bonds.

### 2.3. Electronic Properties

#### 2.3.1. Electrostatic Potential Maps

Molecular electrostatic potential (MEP) surfaces are valuable tools for predicting the reactivity and interaction patterns of bioactive compounds in biological environments [38]. Previous studies have shown that regions of a molecule exhibiting pronounced electrostatic potential often correlate with sites involved in electron donor–acceptor interactions [39].

**Figure 3:**
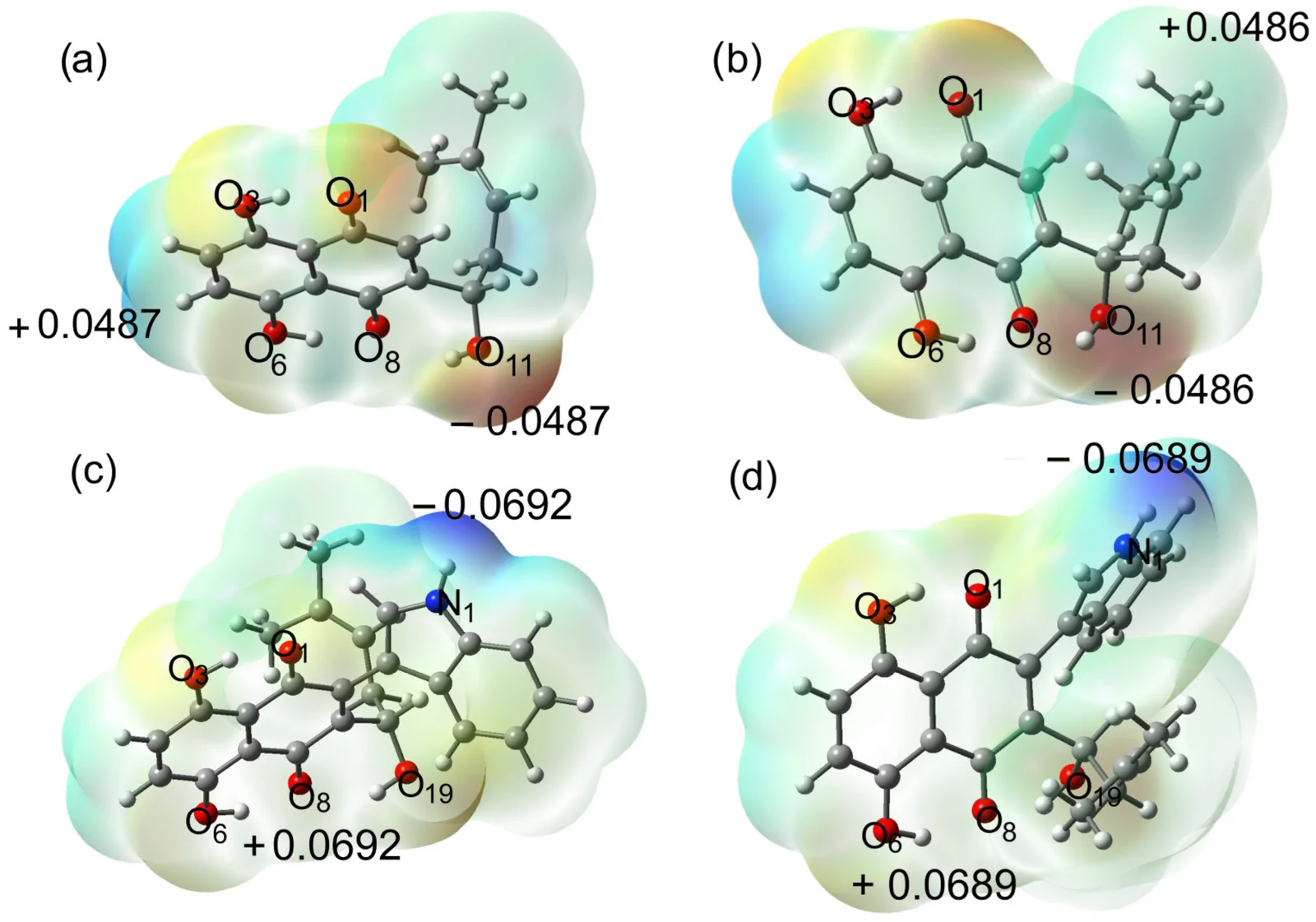
Electrostatic potential maps of (**a**) **1**, (**b**) **2**, (**c**) **3**, and (**d**) **4**.

The MEP surfaces of compounds **1**–**4** were computed at the B3LYP/6-311++G(d,p) level of theory; thus, the resulting maps display a color gradient ranging from deep blue, corresponding to regions of the most positive electrostatic potential, to deep red, representing regions of the most negative electrostatic potential, as shown in Figure 3.

The MEP analysis reveals that electron density is localized on the carbonyl oxygen atoms of the quinone ring [3,20,40]. Also, a negative electrostatic potential is observed on the hydroxyl oxygen atoms. This distribution has been reported previously [20,41].

Conversely, the blue regions correspond to the most electron-deficient sites, which are primarily associated with the hydrogen atom of the indole NH group. These positively polarized regions are expected to participate in hydrogen-bonding and other noncovalent interactions with amino acid residues in biological receptors, thereby contributing to the stabilization of ligand–receptor complexes [38,39].

Complementarily, atomic charges were calculated using the Natural Population Analysis (NPA) method (see Table S4, Supplementary Materials). In all compounds, the oxygen atoms exhibited the most negative atomic charges, ranging from −0.707 e^−^ on O1 to −0.759 e^−^ on O11 for compounds **1** and **2**, and from −0.703 e^−^ on O1 to −0.757 e^−^ on O19 for compounds **3** and **4**. In contrast, the hydrogen atoms of the hydroxyl groups displayed positive atomic charges ranging from +0.475 to +0.502 e^−^ in all four molecules. The nitrogen atoms in compounds **3** and **4** exhibited an average atomic charge of approximately −0.39 e^−^.

These results are consistent with the MEP shown in Figure 3. The highly negative charges localized on the oxygen atoms identify these sites as favorable hydrogen-bond acceptors, whereas the positively polarized hydroxyl hydrogen atoms are likely to act as hydrogen-bond donors. Together, these features suggest that the quinone and indole moieties may participate in hydrogen-bonding interactions with amino acid residues within the binding sites of biological targets.

#### 2.3.2. Molecular Orbitals

The reactivity–stability of the studied molecules was evaluated using the energy of the frontier molecular orbitals. The highest occupied molecular orbital (HOMO) represents the electron-donating character of the molecule, whereas the lowest unoccupied molecular orbital (LUMO) represents its electron-accepting character [42]. Based on the HOMO and LUMO energies, the LUMO–HOMO energy gap (ΔE_LUMO–HOMO_) was calculated as an indicator of chemical reactivity and biological activity. Indolyl quinones have been reported to exhibit cytotoxic activity against breast cancer cells, and several studies have suggested that this activity is related, at least in part, to molecular reactivity descriptors, including the ΔE_LUMO–HOMO_ [3,17,20].

The calculated frontier molecular orbital energies and ΔE_LUMO–HOMO_ are summarized in Table 1. Based on the ΔE_LUMO–HOMO_ values, the predicted order of chemical reactivity is **4** > **3** > **1** > **2**, indicating that indolylshikonin (**4**) is the most reactive compound. As expected for enantiomeric compounds, molecules **1** and **2**, as well as **3** and **4**, exhibit very similar ΔE_LUMO–HOMO_ values. Nevertheless, compounds **3** and **4** display slightly smaller energy gaps than compounds **1** and **2**, suggesting a modest increase in chemical reactivity following indole substitution.

**Figure 4:**
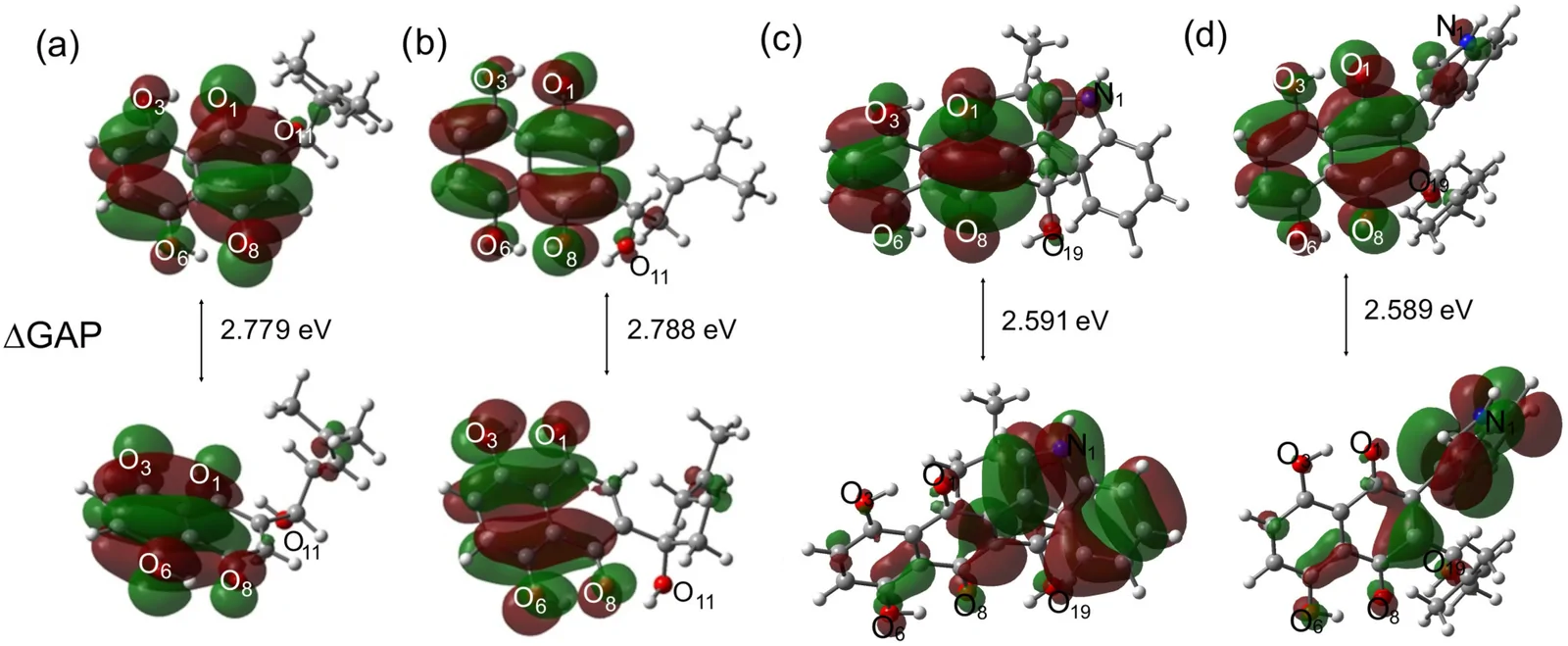
Molecular orbitals HOMO-LUMO distribution of (**a**) **1**, (**b**) **2**, (**c**) **3**, and (**d**) **4**. Most relevant atoms enumeration (shown in Figure 1) are labelled to facilitate their visualization and comparison.

In addition to calculating the ΔE_LUMO–HOMO_ gap, the spatial distributions (molecular regions) of the frontier molecular orbitals were analyzed. The corresponding molecular orbital isosurfaces are shown in Figure 4.

For compounds **1** and **2**, the HOMO is predominantly localized over the quinone ring, with only a minor contribution from the O11 oxygen atom in compound **1**. In both molecules, the LUMO is primarily localized on the aromatic quinone ring. In contrast, for derivatives **3** and **4**, the HOMO is localized over the entire indole ring and the adjacent portion of the quinone ring. In contrast, the LUMO extends over the entire quinone ring and the pyrrolic portion of the indole moiety (Figure 4).

#### 2.3.3. Reactivity Analysis

Table 2 summarizes the neutral, cationic, and anionic energies calculated using DFT, from which the global reactivity descriptors were derived. Several physicochemical descriptors have been proposed to characterize the chemical behavior of molecules, including ionization energy, electron affinity, chemical hardness (η), and the electrophilicity index (ω) [43]. Among these, chemical hardness and electrophilicity are widely used to describe molecular stability and reactivity within the conceptual DFT framework [44]. According to the literature, larger ΔE_LUMO–HOMO_ values are generally associated with harder molecules and greater kinetic stability [43]. The calculated hardness values increased in the following order: **3** (2.594 eV) = **4** (2.594 eV) < **2** (3.319 eV) < **1** (3.320 eV). This trend is consistent with the maximum hardness principle, which states that, under comparable conditions, the most stable molecular arrangement tends to exhibit the highest hardness [45,46]. Accordingly, compound **1** is predicted to be the hardest and, therefore, the least chemically reactive molecule in the series. Conversely, compounds **3** and **4**, corresponding to the indole derivatives, exhibited the smallest ΔE_LUMO–HOMO_ and the lowest hardness values, suggesting greater chemical reactivity with the possibility of generating biological activity. These electronic features may favor interactions with biological targets and could contribute to their potential biological activity [47]. Overall, the introduction of the indole moiety resulted in a decrease in both the ΔE_LUMO–HOMO_ gap and the chemical hardness.

The electrophilicity index (ω) measures the tendency of a molecule to accept electrons. Higher ω values indicate stronger electrophilic character, whereas lower values are generally associated with greater nucleophilic character [48]. The calculated electrophilicity values increase in the following order: **1** (3.483 eV) < **2** (3.490 eV) < **4** (4.414 eV) = **3** (4.414 eV). Accordingly, compound **3** exhibits the highest electrophilic character, whereas between the original compounds **1** and **2**, and the indole compounds **3** and **4** display very similar electrophilicity values.

Electronegativity (χ) is a useful empirical concept for describing the chemical reactivity of molecules, as it quantifies their tendency to attract electrons [49]. According to Mulliken, electronegativity is defined as the average of the ionization potential (IP) and the electron affinity (EA), χ = ½(IP + EA) [50]. The calculated electronegativity values ranged from 4.787 to 4.809 eV, with compounds **1**,**2** exhibiting the highest value, indicating the greatest tendency to attract electrons. Electronegativity is directly related to the electronic chemical potential (μ), since μ = −χ within the framework of conceptual DFT. The electronic chemical potential describes the tendency of a molecular system to exchange electron density with its surroundings in the ground state [51]. Molecules with more negative μ values exhibit a greater tendency to accept electron density, whereas less negative μ values indicate a relatively greater tendency to donate electron density [52]. The calculated chemical potential values increased in the following order for donating electrons: **1** (−4.809 eV) < **2** (−4.810 eV) < **3** (−4.786 eV) = **4** (−4.786 eV). Thus, compound **1** possesses the highest negative electronic chemical potential and the highest electronegativity, whereas compounds **3** and **4** exhibit the highest chemical potential values (less negative) in the series.

Regarding the ionization potential (IP), all compounds exhibited positive values ranging from 7.38 to 8.13 eV. The calculated IP values increased in the following order: **3** (7.380 eV) = **4** (7.380 eV) < **1** (8.130 eV) < **2** (8.131 eV). These results indicate that the indole derivatives (**3**–**4**) require less energy to lose an electron and are therefore more prone to electron donation than the parent naphthoquinones [53]. Finally, the electron affinity (EA) was calculated as the energy difference between the neutral molecule and its corresponding anionic species. The calculated EA values increased in the following order: **1** (1.480 eV) < **2** (1.481 eV) < **3** = **4** (2.191 eV), indicating that compounds **3**–**4** exhibit the greatest tendency to accept an additional electron, whereas compound **1** shows the lowest electron affinity.

Finally, compounds **1**–**2** are predicted to be the best electron acceptors because they exhibit the highest electronegativity (χ) and the most negative chemical potential (μ). In contrast, compounds **3** and **4** are predicted to be the most chemically reactive because they display the lowest chemical hardness (η) and the smallest ΔE_LUMO–HOMO_. However, this does not imply that they are the best electron acceptors. Rather, their lower hardness and smaller energy gap indicate that they require less energy to reorganize their electronic structure and, consequently, can participate more readily in charge-transfer processes.

### 2.4. Spectroscopic Properties Analysis

#### 2.4.1. Infrared Spectrophotometry

**Figure 5:**
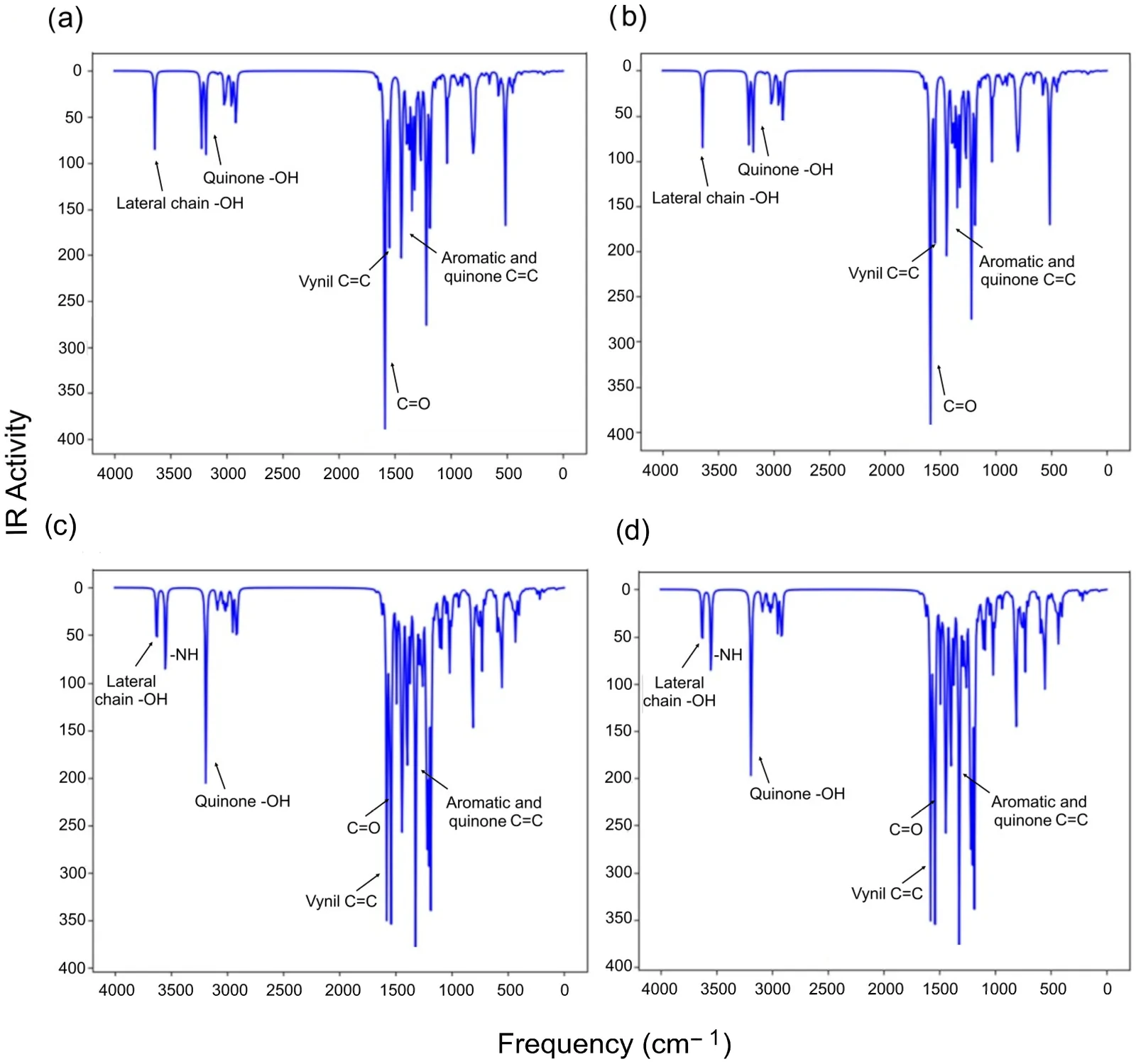
Calculated vibration spectra for the target compounds, computed at B3LYP/6-311++G(d,p) level of theory: (**a**) **1**, (**b**) **2**, (**c**) **3**, and (**d**) **4**.

The spectroscopic properties of the most stable conformers were theoretically predicted in the gas phase. The calculated infrared spectra showed that compounds **1** and **2**, each containing 37 atoms, exhibited 105 normal vibrational modes, whereas compounds **3** and **4**, each containing 51 atoms, exhibited 147 normal vibrational modes. The most relevant theoretical and experimental infrared wavenumbers (available for compounds **1** and **2**) are summarized in Figure 5 and Table S5 of the Supplementary Materials [7]. The calculated harmonic frequencies were scaled using a factor of 0.9679 before comparison with the available experimental data.

The infrared spectral analysis focused on the characteristic vibrational modes of the aromatic, quinone, and vinylic C=C bonds; the carbonyl (C=O) groups; the hydroxyl (O–H) groups; and the additional vibrational modes associated with the indole moiety in compounds **3** and **4**. Overall, the theoretical and experimental vibrational frequencies for compounds **1** and **2** showed good agreement. The largest deviations were observed for the aromatic and quinone C=C stretching vibrations; however, in most cases, the differences between the calculated and experimental wavenumbers were less than 3%.

The most characteristic vibrational bands were associated with the quinone carbonyl (C=O) groups, which appeared between 1638 and 1586 cm^−1^ for compounds **1** and **2**. A slight shift was observed for the indole derivatives, with the corresponding carbonyl stretching bands appearing between 1636 and 1584 cm^−1^ for compounds **3** and **4**. Nevertheless, these bands remained within the same characteristic spectral region.

Another important absorption band corresponded to the stretching vibration of the vinyl C=C bond in the side chain. This vibration was calculated at approximately 1669 cm^−1^ for compounds **1** and **2** and between 1670 and 1671 cm^−1^ for compounds **3** and **4**.

In addition, the O–H stretching vibrations were identified according to their structural environment. The hydroxyl group of the side chain exhibited stretching frequencies at approximately 3639–3636 cm^−1^ for compounds **1** and **2** and around 3626 cm^−1^ for compounds **3** and **4**. In contrast, the quinone hydroxyl group exhibited lower stretching frequencies because of intramolecular hydrogen bonding, appearing between 3224 and 3184 cm^−1^ for compounds **1** and **2**, and between approximately 3192 and 3186 cm^−1^ for compounds **3** and **4**.

Finally, an additional band was observed at approximately 3547 cm^−1^ in the calculated spectra of compounds **3** and **4**, corresponding to the N–H stretching vibration of the indole moiety. This characteristic absorption, together with the vibrational bands associated with the quinone and indole aromatic regions, represents the main spectroscopic difference between shikonin, alkannin, and their corresponding indole derivatives.

These results for **1** and **2** demonstrated a good correlation between the theoretical and experimental values, allowing appropriate band assignments. Related to **3** and **4**, the studied molecules are new and are in progress in our research laboratory.

#### 2.4.2. Nuclear Magnetic Resonance ^1^H and ^13^C in Gas Phase and Solvent Effect

**Figure 6:**
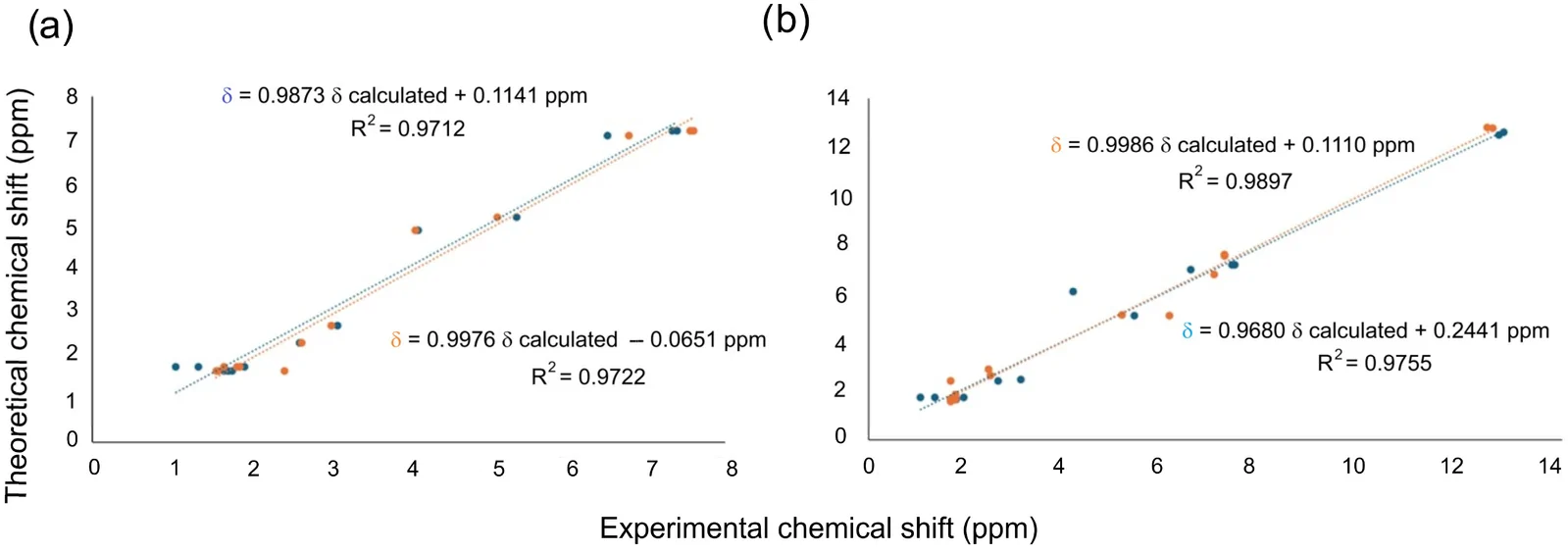
Linear regression analysis of the ^1^H NMR chemical shifts between the theoretical values calculated at the B3LYP/6-311++G(d,p) level and the experimental data for (**a**) compound **1** and (**b**) compound **2**.

Theoretical ^1^H and ^13^C nuclear magnetic resonance (NMR) chemical shifts were calculated for the target molecules, **1**–**4**, in both the gas phase and chloroform solvent. The calculations were performed using the B3LYP functional with the 6-311++G(d,p) basis set and the Gauge-Independent Atomic Orbital (GIAO) method. Figure 6 (^1^H NMR, gas phase and solvent) and 7 (^13^C NMR, gas phase and solvent) present the linear regression analyses correlating the calculated and experimental chemical shifts for **1** and **2** [54,55,56]. The calculated ^1^H and ^13^C chemical shifts for compounds obtained in both the gas phase and chloroform solvent were compared with the available experimental data (Tables S6 and S7), highlighting that no experimental NMR data have been reported yet for the indole derivatives (**3** and **4**), impeding a similar comparison.

The correlation between the calculated and experimental ^1^H NMR chemical shifts in the gas phase is presented in Figure 6 (blue trendline). For compound **1,** the linear regression analysis yielded a correlation coefficient (R^2^) of 0.9712, a standard deviation of 2.3062, and a standard error of 0.4171 ppm. Similarly, compound **2** exhibited a correlation coefficient (R^2^) of 0.9755, a standard deviation of 3.8101, and a standard error of 0.6230 ppm. The corresponding regression equations were δ_theoretical_ = 0.9873δ_calculated_ + 0.1141 ppm for compound **1** and δ_theoretical_ = 0.9680δ_calculated_ + 0.2441 ppm for compound **2**.

By contrast, the correlation between the ^1^H NMR chemical shifts calculated in chloroform solvent and the experimental values is also presented in Figure 6 (orange trendline). Compound **1** showed a slightly higher regression coefficient than that obtained in the gas phase (0.9722), with a standard deviation of 2.2951 and a typical error of 0.4100 ppm. In contrast, compound **2** exhibited a regression coefficient of 0.9976, a standard deviation of 3.7788, and a typical error of 0.4046 ppm. The corresponding regression equations are δ_theoretical_ = 0.9976δ_calculated_ − 0.0651 ppm for **1** and δ_theoretical_ = 0.9985δ_calculated_ + 0.1110 ppm for **2**. These results indicate that both compounds exhibit improved regression coefficients when solvent effects are included in the calculations, highlighting the importance of accounting for the solvent phase in DFT calculations [57].

**Figure 7:**
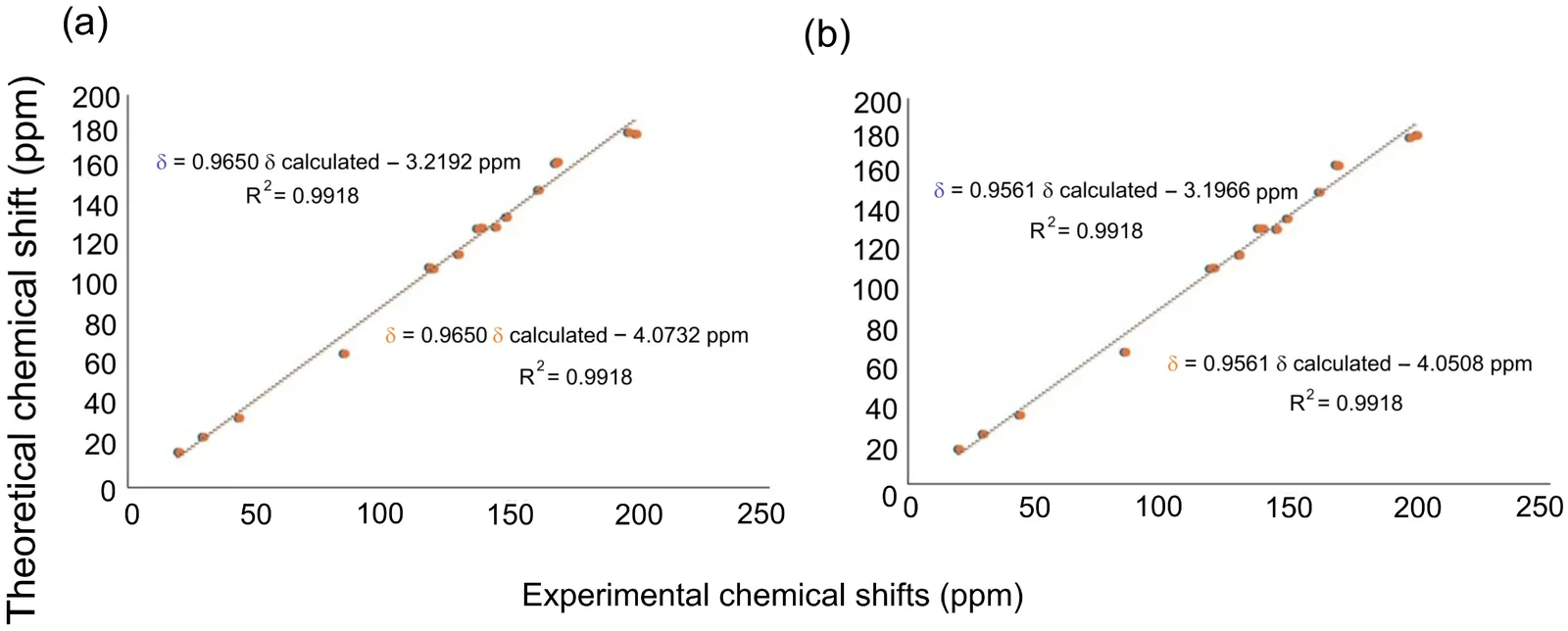
Linear regression analysis of the ^13^C NMR chemical shifts between the theoretical values calculated at the B3LYP/6-311++G(d,p) level and the experimental data for (**a**) compound **1** and (**b**) compound **2**.

The linear regression analysis of the ^13^C NMR chemical shift data is presented in Figure 7, where the blue and orange trendlines correspond to the gas- and solvent-phase calculations, respectively. Compound **1** exhibited an excellent regression coefficient (R^2^ = 0.9918), with a standard deviation of 53.056 and a typical error of 5.1140 ppm. Similarly, compound **2** showed the same regression coefficient (R^2^ = 0.9918), with a standard deviation of 53.0563 and a typical error of 5.1212 ppm. The corresponding regression equations are δ_theoretical_ = 0.9650δ_calculated_ − 3.2192 ppm for **1** and δ_theoretical_ = 0.9561δ_calculated_ − 3.1966 ppm for **2**.

By contrast, the correlation between the ^13^C NMR chemical shifts calculated in chloroform solvent and the experimental values is also presented in Figure 7. Compound **1** exhibited the same regression coefficient (0.9918) as in the gas phase, with a standard deviation of 53.0908 ppm and a typical error of 5.1140 ppm. Likewise, compound **2** showed the same regression coefficient (0.9918), with a standard deviation of 53.0906 and a typical error of 5.1213 ppm. The corresponding regression equations are δ_heoretical_ = 0.9650δ_calculated_ − 4.0732 ppm for **1** and δ_theoretical_ = 0.9651δcalculated − 4.0508 ppm for **2**. For ^13^C NMR chemical shifts, the regression coefficients remained unchanged, indicating that the inclusion of solvent effects produced no significant differences compared with the gas-phase DFT calculations for these molecules.

Thus, the results for **1** and **2** demonstrated a good correlation between the theoretical and experimental values, allowing appropriate chemical shift assignments. Related to **3** and **4**, the studied molecules are new and are in progress in our research laboratory.

### 2.5. Hydrogen Bonds Evaluation

**Figure 8:**
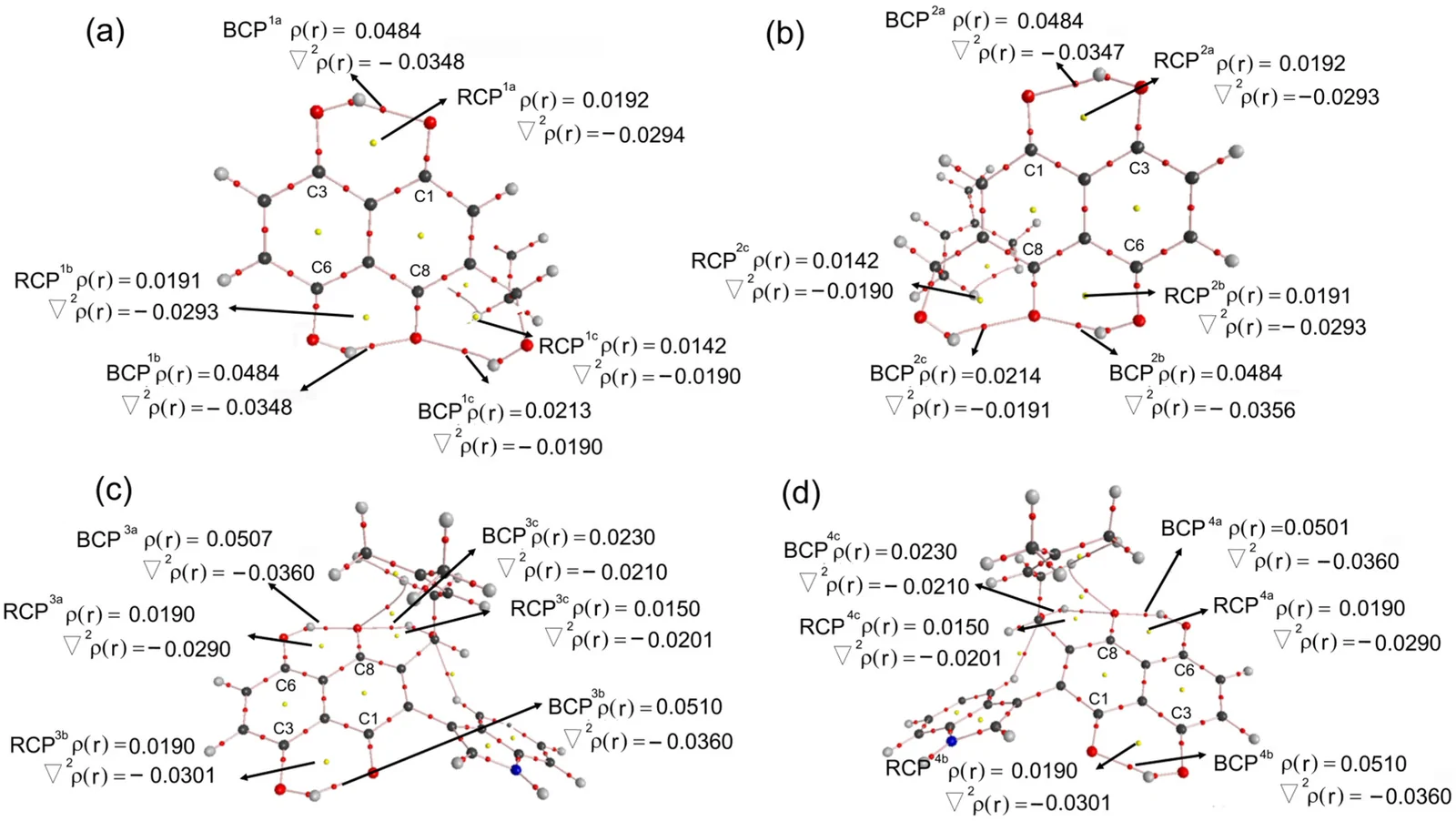
Molecular graphs of compounds **1**–**4**. Electron density (ρ) and its Laplacian (∇^2^ρ) at the bond critical points (BCPs) and ring critical points (RCPs) characterizing the intramolecular hydrogen-bond interactions: (**a**) compound **1,** (**b**) compound **2,** (**c**) compound **3**, and (**d**) compound **4**.

It is well known that hydrogen-bond (HB) interactions are associated with characteristic changes in molecular geometry and structural stability, giving rise to different criteria for assessing the strength of these interactions [58]. To this end, Bader developed the Quantum Theory of Atoms in Molecules [59], which provides a rigorous quantum-mechanical partitioning of a molecule into its constituent atoms and yields a variety of useful topological descriptors. The stable critical points of the electron density, ρ(r), are classified into four categories: maxima correspond to nuclear attractors, minima correspond to cage critical points (CCPs), and saddle points correspond to bond critical points (BCPs) and ring critical points (RCPs) [60]. Accordingly, a topological analysis was performed to characterize the intramolecular interactions. Contour plots of the electron density calculated from the B3LYP/6-311++G(d,p) wavefunctions of **1**–**4** are presented in Figure 8.

Bond critical points (BCPs) were identified for compounds **1**–**4**, confirming the presence of intramolecular hydrogen-bond interactions involving the carbonyl groups (C1 and C8) and the hydroxyl groups located on the naphthoquinone ring and the side chain. The corresponding electron density, ρ(r), and Laplacian, ∇^2^ρ(r), values at each BCP are summarized in Table S8 of the Supplementary Materials.

Several topological criteria for the existence of hydrogen bonds have been proposed [60,61]. Koch and Popelier established that a bond critical point (BCP) must be located within the H···A contact region, which topologically demonstrates an increase in the electron density and confirms the existence of a hydrogen bond. According to these authors, the electron density at the BCP should range from 0.002 to 0.040 e.a.u.−^3^ [60,61]. For the compounds studied here, the electron density values are slightly higher than the proposed range, except for some BCPs in compounds **3** and **4**.

Hydrogen bonds can be further classified as closed-shell interactions (electrostatic in nature) when low ρ(r) and positive ∇^2^ρ(r) values are observed, whereas shared-shell interactions are characterized by higher ρ(r) and negative ∇^2^ρ(r) values [62]. Nevertheless, a negative Laplacian at the bond critical point has also been associated with stronger hydrogen bonds exhibiting partial covalent character due to increased electron sharing [63]. Therefore, the hydrogen bonds identified in compounds **1**–**4** may present a significant degree of covalent contribution.

Moreover, because of the hydrogen-bond interactions, an associated ring critical point (RCP) was identified for each analyzed structure. The topological analysis revealed the presence of RCPs located at the center of the rings generated by the intramolecular hydrogen bonds. The corresponding electron density (ρ(r)) and Laplacian (∇^2^ρ(r)) values for compounds **1**–**4** are summarized in Table S9, Supplementary Materials.

RCP values can be explained by previous reports indicating that low and negative values of ρ(r) and ∇^2^ρ(r), respectively, reflect ring stabilization through electronic delocalization. This stabilization is due to the ring being formed by a favorable geometry and supported by electron redistribution [64,65].

### 2.6. Chemoinformatic Evaluation

The studied compounds exhibited potential cytotoxic effects in cancer cells; therefore, their bioactivity, toxicity risk, and key physicochemical properties were predicted using Way2Drug (PASS Online) [66], SwissADME [67], ProTox 3.0 [68], and Osiris Property Predictor [69]. The first analysis was performed using the PASS server, which enables the prediction of different types of pharmacological activities. This tool is based on a modified naïve Bayes algorithm and was applied because it has demonstrated robustness, providing reliable predictions of various biological activities based on the structural formula of compounds [70]. In this context, molecules **1**–**4** were analyzed using the PASS server. It is important to note that this platform does not distinguish between enantiomeric structures; consequently, compounds **1** and **2** showed identical biological predictions, whereas compounds **3** and **4** exhibited the same predicted activities. The results of the PASS analysis are summarized in Table 3.

The PASS predictor provides results as probabilities, where Pa represents the probability of being active, and Pi corresponds to the probability of being inactive. In this context, a Pa value higher than 0.7 indicates a higher probability of exhibiting biological activity.

The bioactivity prediction of the studied molecules showed that compounds **1**–**4** exhibited a probability of apoptosis agonist activity, with Pa values of 0.754 for the naphthoquinones (**1** and **2**) and 0.654 for the indolyl naphthoquinones (**3** and **4**). Additionally, the studied compounds showed potential antineoplastic activity, with Pa values of 0.710 for compounds **1** and **2** and 0.605 for compounds **3** and **4**. Furthermore, naphthoquinones **1** and **2** showed a probability of activity against breast and lung cancer.

Complementary to the PASS server results, toxicological and physicochemical properties of the studied compounds were evaluated to further assess their potential bioactive properties. Since the molecules exhibited potential effects on human cancer cells, toxicity risk prediction and the analysis of relevant physicochemical parameters were performed using the Osiris Property Explorer [69]. The corresponding results are summarized in Table 4.

The toxicity risk prediction indicated that compounds **3** and **4** exhibited the lowest risk of undesirable effects, showing no predicted risks of mutagenicity, tumorigenicity, irritant effects, or reproductive effects. In contrast, compounds **1** and **2** exhibited a high risk of irritant effects. These results suggest that this risk is associated with the presence of the α,β-unsaturated quinone moiety at the C10 position, which may explain why compounds **3** and **4** do not exhibit this predicted effect. The predicted toxicity risks and the corresponding physicochemical properties are summarized in Table 4.

Regarding molecular weight, all the studied compounds had values between 160 and 500 g/mol. Additionally, compounds **1**–**4** exhibited no more than five hydrogen bond donors (HBDs) and fewer than ten hydrogen bond acceptors (HBAs), in agreement with Lipinski’s Rule of Five [72].

**Figure 9:**
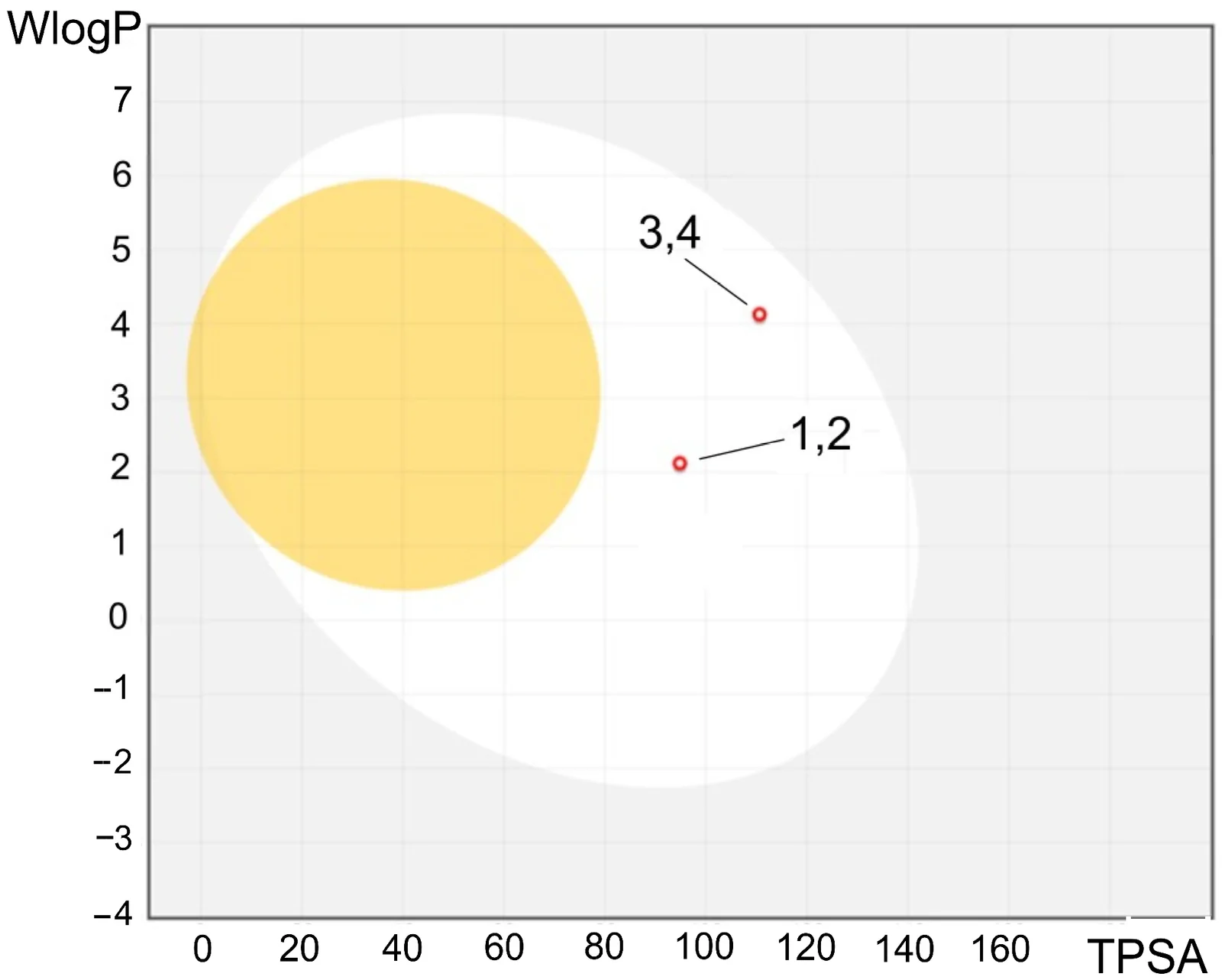
Boiled egg diagram of compounds **1**–**4**.

The physicochemical properties of the compounds were also estimated, including LogP, the logarithm of the partition coefficient between *n*-octanol and water, which describes molecular hydrophobicity. The calculated LogP values ranged from 0.42 to 1.31 for the studied compounds (<5), according to Lipinski’s MLogP consideration. WLogP can also describe high gastrointestinal absorption for all compounds, favorable when oral administration is needed. Figure 9 displays a boiled egg representation, in which the lipophilicity and polarity of small molecules are applied, relating the white zone as the possibility of being passively absorbed by the gastrointestinal tract and the yellow zone as the location where molecules can permeate through the blood–brain barrier [74].

Furthermore, another important descriptor of absorption is the topological polar surface area (TPSA), which is associated with intestinal absorption, oral bioavailability, Caco-2 permeability, and blood–brain barrier (BBB) penetration [75]. The TPSA values for compounds **1** and **2** were identical (94.83 Å^2^), whereas compounds **3** and **4** exhibited TPSA values of 110.62 Å^2^.

To complement the physicochemical characterization and further evaluate the drug-like potential of the studied compounds, predictions of their absorption, metabolism, and excretion properties were performed. The results indicated that compounds **1**–**4** have a high probability of human intestinal absorption. In contrast, none of the compounds was predicted to penetrate the blood–brain barrier.

P-glycoprotein (P-gp) is one of the major ABC transporters. It is widely distributed in various tissues and plays a key role in the ATP-dependent efflux of xenobiotics against concentration gradients. P-glycoprotein is also involved in the transport of a wide range of small molecules across biological membranes. This transporter is highly expressed in multidrug-resistant cancer cells, and its inhibition has been proposed as a strategy to overcome multidrug resistance [76]. Compounds **3** and **4** were predicted to be P-gp substrates. In contrast, compounds **1** and **2** were predicted to be substrates of CYP2C9, one of the major cytochrome P450 (CYP450) isoforms involved in drug metabolism [77].

### 2.7. Molecular Coupling Analysis

Molecular docking studies of compounds **1**–**4** were performed against three proteins involved in cancer-related processes: PARP-1, COX-2, and HDAC2 (Table S10, Supplementary Materials). The docking protocol was validated by redocking the co-crystallized ligand into the binding site of each protein and subsequently calculating the root-mean-square deviation (RMSD). RMSD values below 2.0 Å were considered indicative of a reliable docking protocol [78].

#### 2.7.1. Analysis of Molecular Coupling and Most Relevant Interactions with PARP-1

**Figure 10:**
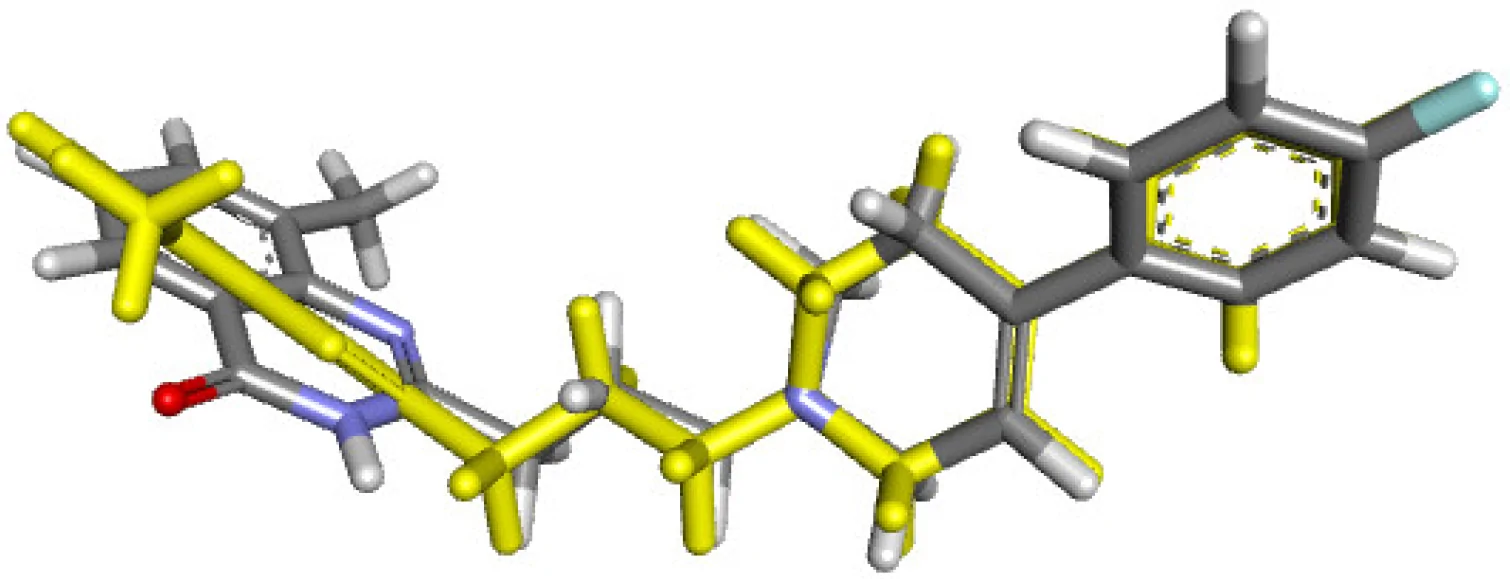
Root-mean-square deviation (RMSD) between the redocked ligand FR257517 (yellow) and the co-crystallized reference ligand (gray), illustrating a reliable docking solution (RMSD ≤ 2.0 Å) for the validation of the PARP-1 docking protocol.

In mammals, members of the PARP family (PARP1–PARP6) share a conserved His–Tyr–Glu catalytic triad within the nicotinamide-binding pocket of the catalytic domain [79], making this region an attractive target for molecular docking studies. The docking protocol for PARP-1 was first validated by redocking the co-crystallized ligand FR257517 into the active site using the same protocol applied to all tested compounds (Figure 10). The resulting root-mean-square deviation (RMSD) between the redocked and co-crystallized ligand poses was 1.1153 Å. RMSD values below 2.0 Å are generally considered indicative of a reliable docking protocol [78].

Following validation of the docking protocol, molecular docking of olaparib was performed as a reference compound because of its well-established inhibitory activity against PARP-1 in several types of cancer [80,81,82].

**Figure 11:**
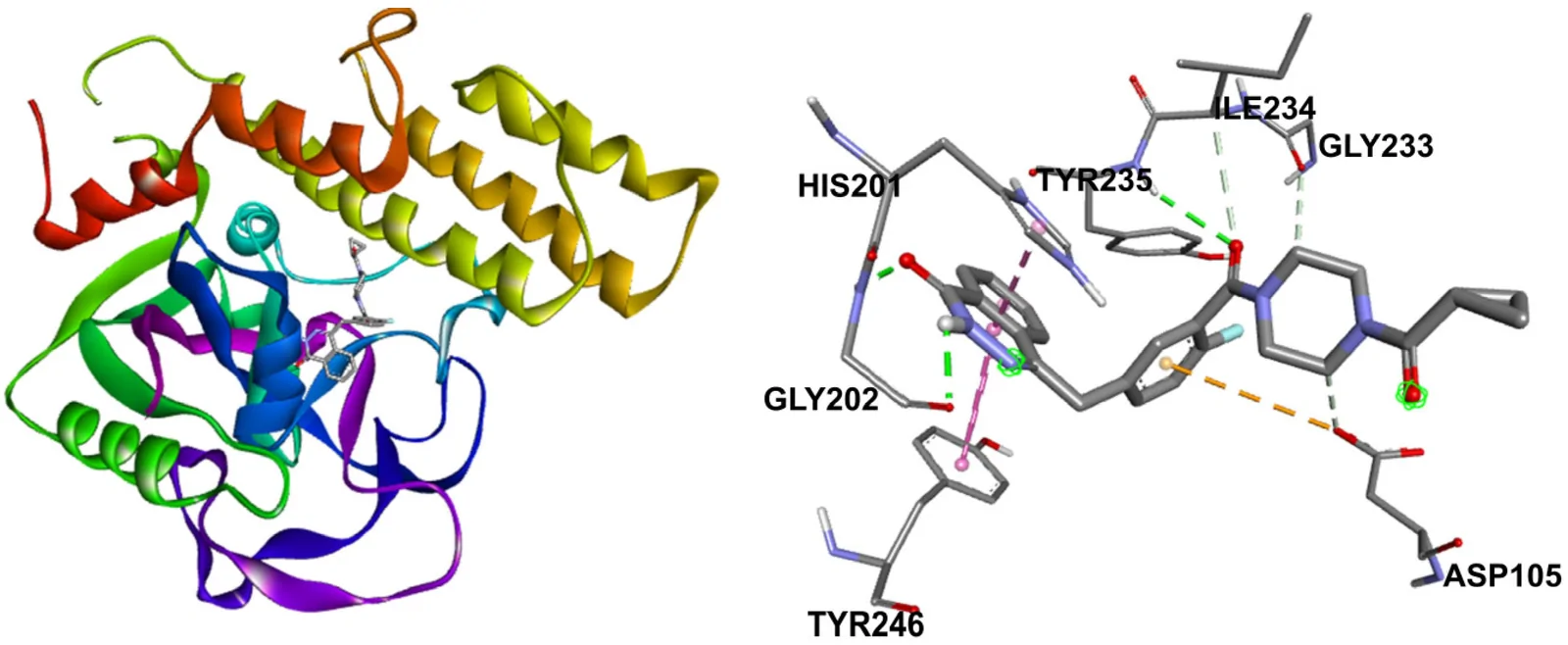
Docking binding mode and main interactions between PARP-1 and olaparib.

**Figure 12:**
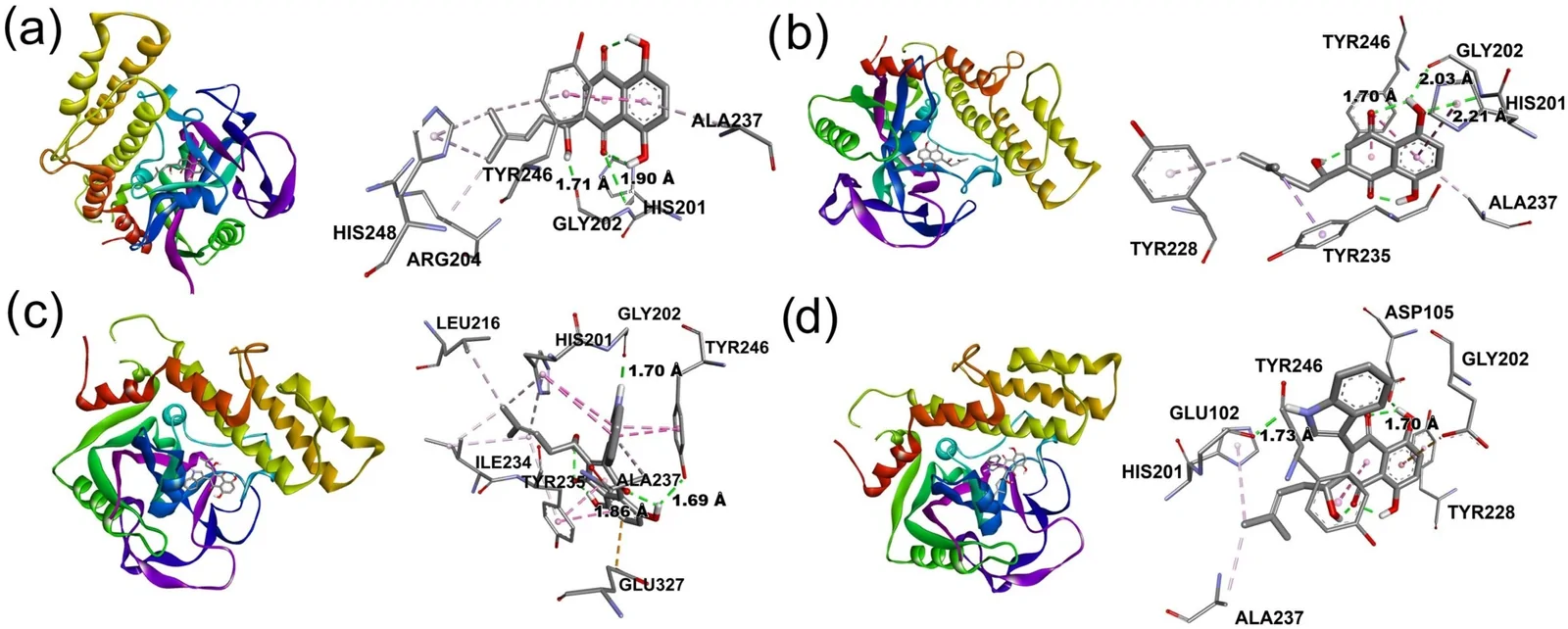
Molecular coupling binding modes and principal interactions with human PARP-1 of (**a**) **1**, (**b**) **2**, (**c**) **3**, and (**d**) **4**.

The active site of PARP-1 corresponds to the catalytic triad, which consists of His201, Tyr235, and Glu327 [83]. The reference inhibitor, olaparib, exhibited a binding energy (ΔG) of −10.23 kcal/mol (Figure 11). Its binding mode included hydrogen bond interactions with Tyr235 and Gly202, as well as π–π stacking interactions with His201 and Tyr246. Compounds **1**–**4** were subsequently docked into the same binding site occupied by olaparib. Among them, compound **1** exhibited the most favorable binding affinity, with a binding energy (ΔG) of −11.44 kcal/mol, surpassing that of olaparib. Two hydrogen bond interactions were observed: one between the carbonyl group of **1** and the peptide backbone of the His201–Gly202 residues, and another between a hydroxyl group of **1** and the carbonyl group of Gly202 (Figure 12).

In contrast, **2** exhibited a binding energy (ΔG) of −7.94 kcal/mol. Nevertheless, it is noteworthy that this compound formed a π–σ interaction with Tyr235 and a π–π stacking interaction with His201.

A marked difference in the binding energies of the alkannin/shikonin enantiomeric pair was observed, suggesting a possible enantioselective interaction with PARP-1. Among the two enantiomers, compound **1** exhibited the highest binding affinity, achieving the most stable binding conformation within the active site, with a ΔG value of −11.44 kcal/mol. Derivative **3** also showed a higher binding affinity for the PARP-1 active site than olaparib, with a ΔG value of −10.85 kcal/mol. Its binding mode involved multiple π–π stacking interactions with His201, a hydrogen bond with the His201–Gly202 peptide backbone, and an anion–π interaction with Gly237, with the latter not being observed for olaparib or any of the other studied compounds.

In comparison, **4** exhibited a ΔG value of −10.54 kcal/mol, representing a substantial improvement in binding affinity over **2** (ΔG = −7.94 kcal/mol). This enhancement is likely associated with the incorporation of the indole moiety, which promoted favorable π–π stacking interactions with His201 and Tyr246.

The calculated global reactivity descriptors provide a rationale for the docking results. While compound **2** exhibited the greatest electron-accepting ability, compounds **3** and **4** showed the highest chemical reactivity because of their lower chemical hardness and smaller ΔE_LUMO-HOMO_ values, facilitating electronic reorganization during ligand–protein recognition. Nevertheless, the highest PARP-1 affinity was achieved by compound **1**, indicating that binding depends not only on electronic reactivity but also on stereochemistry and the complementarity of ligand–protein interactions.

#### 2.7.2. Analysis of Molecular Coupling and Most Relevant Interactions with COX-2

**Figure 13:**
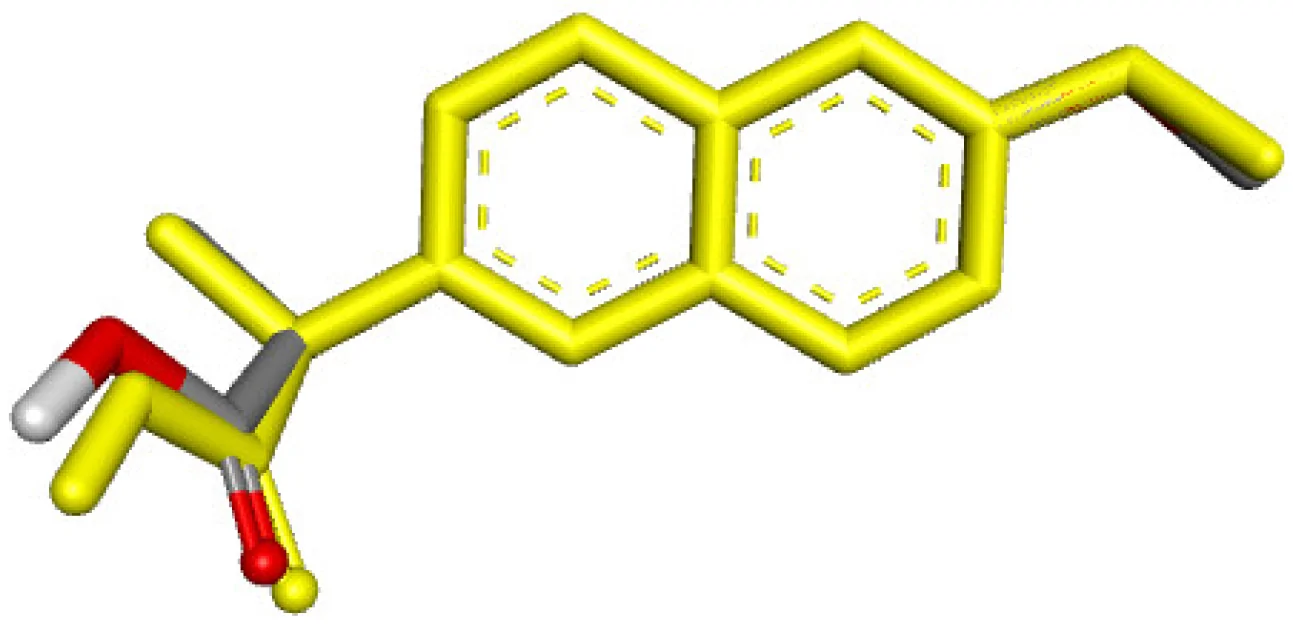
RMSD for co-crystallized ligand naproxen (yellow) with respect to the reference ligand at the crystal structures (gray) to illustrate a good docking solution (RMSD ≤ 2.0 Å).

Cyclooxygenase-2 (COX-2) is an enzyme involved in the biosynthesis of prostaglandins and thromboxane, which regulate biological processes such as inflammation, angiogenesis, and cell proliferation. COX-2 is overexpressed in a variety of malignant tissues and tumors, making it an attractive therapeutic target in cancer treatment [84]. The COX-2 active site consists of a predominantly hydrophobic binding pocket extending from Arg120 to Tyr385. This pocket is relatively large and includes the amino acid residues Val523, Val434, Leu503, and Arg513, which contribute to a hydrophilic region capable of accommodating both polar and nonpolar ligands [85]. The docking protocol was validated by redocking the co-crystallized ligand naproxen into the original protein structure, yielding an RMSD value of 0.2910 Å (Figure 13), which confirms the reliability of the docking protocol.

**Figure 14:**
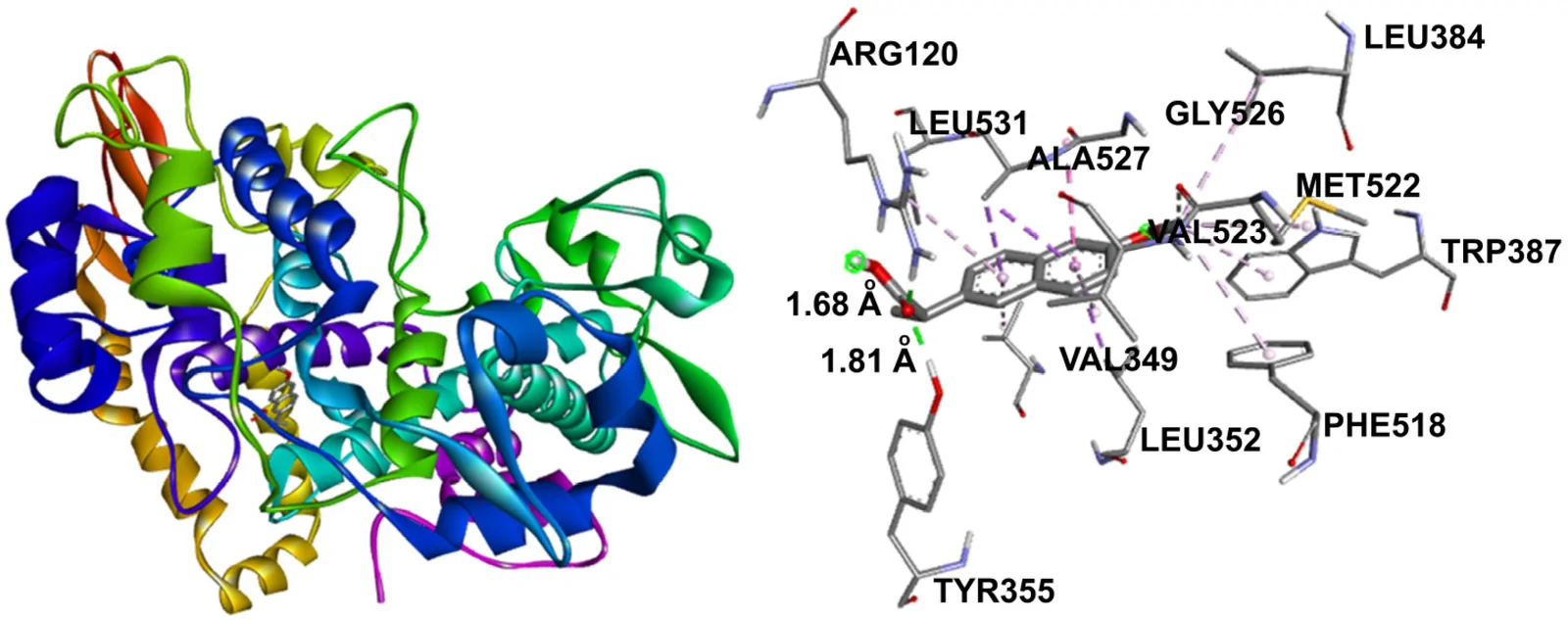
Docking binding mode and main interactions between COX-2 and naproxen.

Naproxen was used as the reference inhibitor because of its well-established inhibitory activity against COX-2 [86]. Its predicted binding pose (Figure 14) exhibited a binding energy (ΔG) of −8.75 kcal/mol (Table S10, Supplementary Materials). The binding mode was consistent with previously reported crystallographic and molecular docking studies [87,88]. Naproxen formed key hydrogen bond interactions with Tyr355 and Arg120, as well as hydrophobic interactions with Leu531, Trp387, Val349, and Leu352. In addition, an interaction with Val523, a residue associated with the larger secondary pocket that contributes to COX-2 selectivity, was observed.

**Figure 15:**
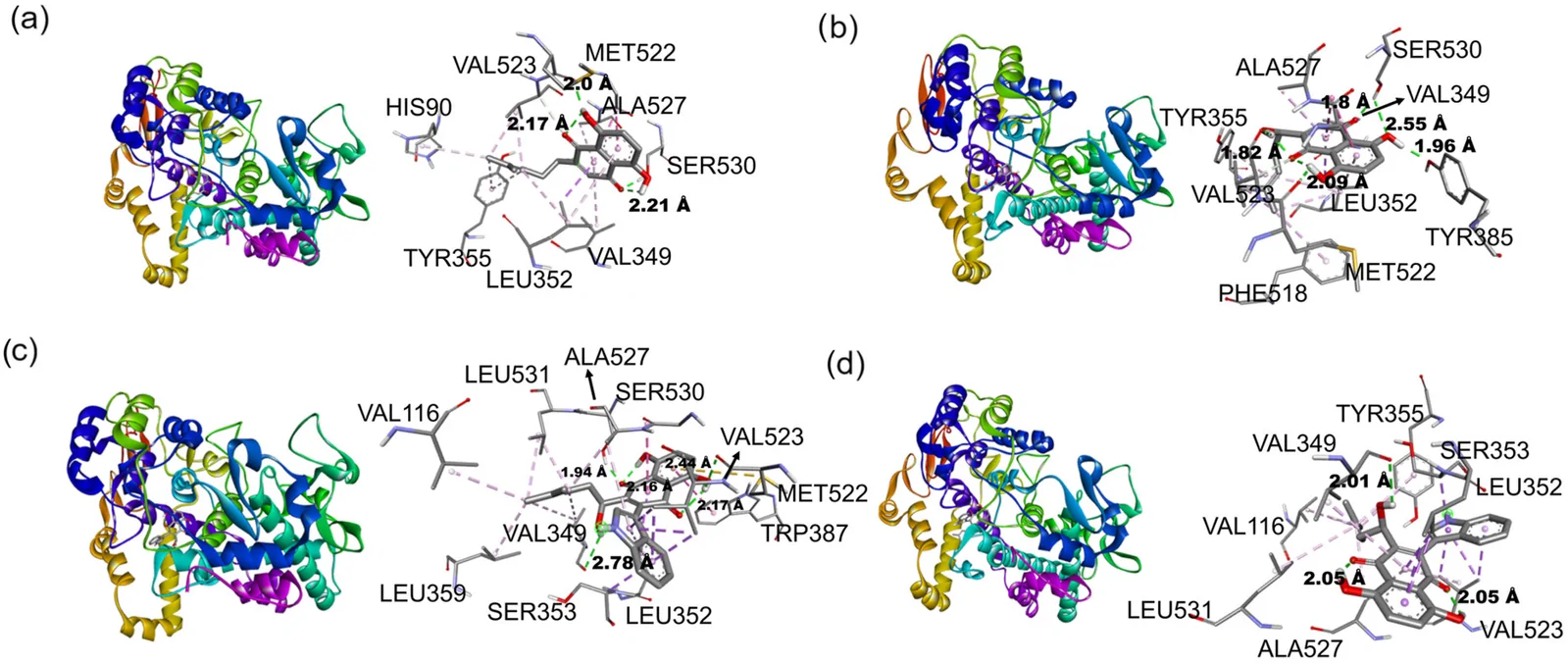
Molecular coupling binding modes and principal interactions with COX-2 of (**a**) **1**, (**b**) **2,** (**c**) **3**, (**d**) **4**.

The molecular docking results for COX-2 showed that all studied compounds exhibited more favorable binding energies than naproxen (ΔG = −8.75 kcal/mol). In addition to their higher predicted binding affinities, the compounds showed several interactions with key residues within the COX-2 binding site, predominantly through hydrophobic contacts and, to a lesser extent, hydrogen bonds. The main ligand–protein interactions for compounds **1**–**4** are summarized in Figure 15 and Table S10 (Supplementary Materials).

Among the evaluated compounds, **1** exhibited the highest predicted binding affinity, with a binding energy (ΔG) of −11.20 kcal/mol, followed by **4** (ΔG = −10.14 kcal/mol). These results suggest that the *S* enantiomer (compound **1**) displays a stronger predicted affinity for the COX-2 binding site than its corresponding indole derivative, compound **3** (ΔG = −9.64 kcal/mol). In contrast, shikonin (compound **2**) exhibited a lower predicted binding affinity (ΔG = −8.44 kcal/mol), whereas its indole derivative (compound **4**) showed a substantially improved binding energy (ΔG = −10.14 kcal/mol). These findings suggest that incorporation of the indole moiety enhances the predicted binding affinity of the *R* enantiomer toward the COX-2 active site.

Compound **1** shared several key interactions with naproxen, including contacts with Tyr355, Val349, Leu352, and Val523. The interaction with Val523 is particularly noteworthy because this residue contributes to the structural basis of COX-2 selectivity. Most of these interactions were hydrophobic, although a hydrogen bond with Val523 was also observed. Similarly, compound **4** interacted with Tyr355, Val349, Leu352 (hydrogen bond), and Val523, indicating a binding mode comparable to that of naproxen and **1**. In addition, **4** also interacted with Leu531 through its hydroxyl substituent, a contact likewise observed for naproxen (Figure 15).

The global reactivity descriptors provide a useful framework for interpreting the COX-2 docking results. Although compounds **3** and **4** exhibited higher predicted chemical reactivity because of their lower chemical hardness and smaller ΔE_LUMO–HOMO_, the highest binding affinity was observed for **1**. These findings indicate that protein recognition depends not only on electronic reactivity but also on stereochemistry and the specific hydrophobic and hydrogen-bonding interactions established within the COX-2 active site.

#### 2.7.3. Analysis of Molecular Coupling and Most Relevant Interactions with HDAC-2

Finally, the interactions of compounds **1**–**4** with HDAC2 were evaluated by molecular docking. Histone deacetylases (HDACs) are a class of Zn^2+^-dependent metalloenzymes that catalyze the deacetylation of lysine residues in histones, thereby regulating chromatin structure and gene expression [89]. HDAC2 has been implicated in several biological processes, including cell proliferation, cell signaling, cancer initiation and progression, inflammation, and the regulation of gene expression [11].

The HDAC2 active site consists of a lipophilic channel (also referred to as the “tube”) and a hydrophobic cavity known as the “foot pocket.” The lipophilic channel is lined by Gly154, Phe155, His183, Phe210, and Leu276, whereas the foot pocket is formed by Tyr29, Met35, Phe114, and Leu144 [89].

**Figure 16:**
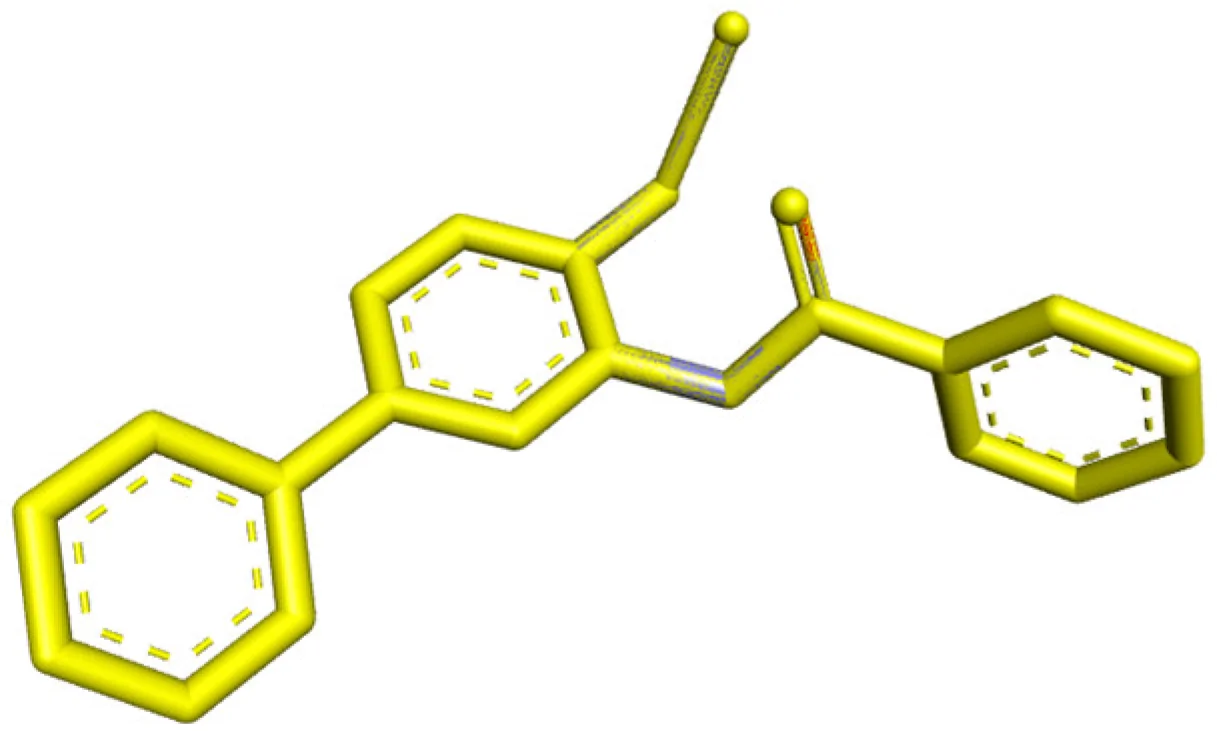
RMSD for co-crystallized ligand KIZ (yellow) with respect to the reference ligand at the crystal structures (gray) to illustrate a good docking solution (RMSD ≤ 2.0 Å).

The docking protocol was validated by redocking the co-crystallized ligand (BIZ) into the original protein structure, yielding an RMSD value of 0.5678 Å (Figure 16), which confirms the reliability of the docking protocol.

**Figure 17:**
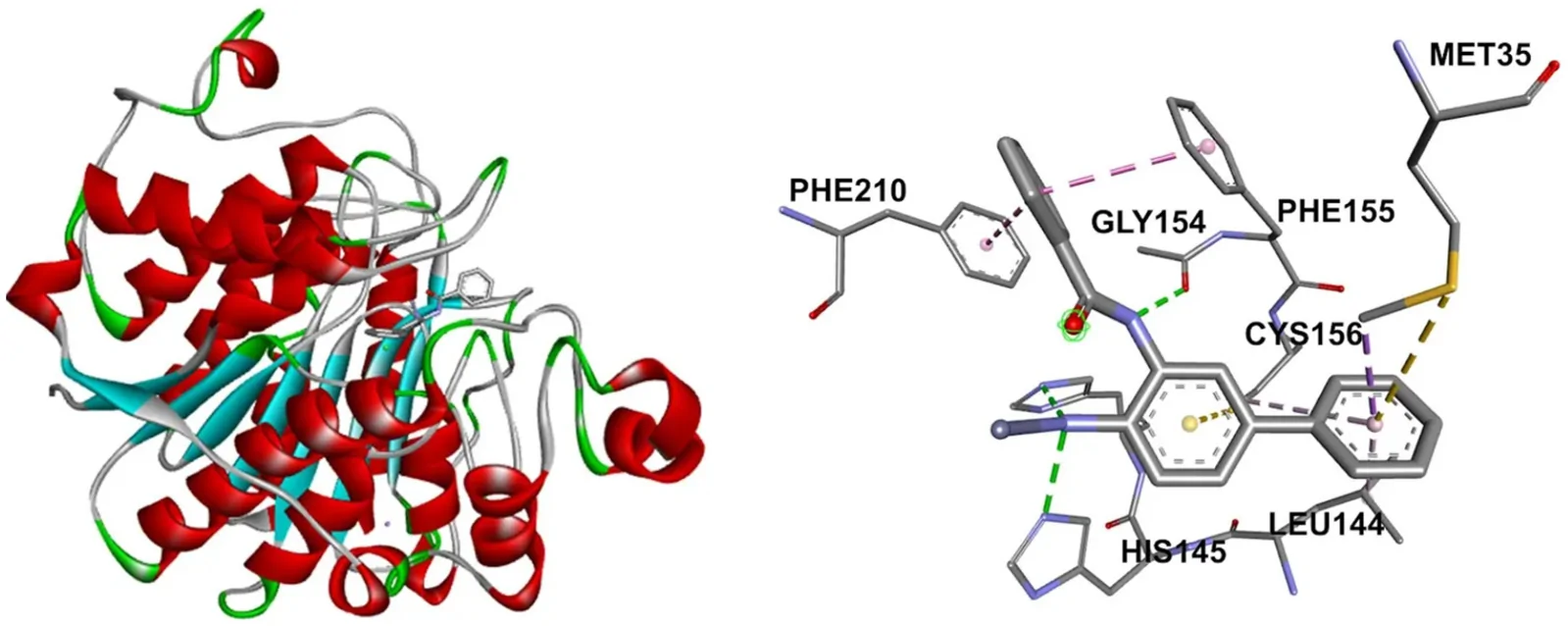
Docking binding mode and main interactions between HDAC2 and BIZ.

The interactions of compounds **1**–**4** with HDAC2 were less favorable than those of the co-crystallized ligand BIZ. None of the evaluated compounds exhibited a more favorable binding free energy than BIZ (ΔG = −8.07 kcal/mol), which has been reported as an HDAC2 inhibitor [89] (Figure 17).

The most representative interactions between BIZ and HDAC2 occurred within the lipophilic channel, where Gly154, Phe155, and Phe210 engaged primarily in hydrophobic interactions and hydrogen bonding. In addition, residues located in the foot pocket, including Met35 and Leu144, established favorable sulfur–π interactions with the aromatic rings of the ligand.

**Figure 18:**
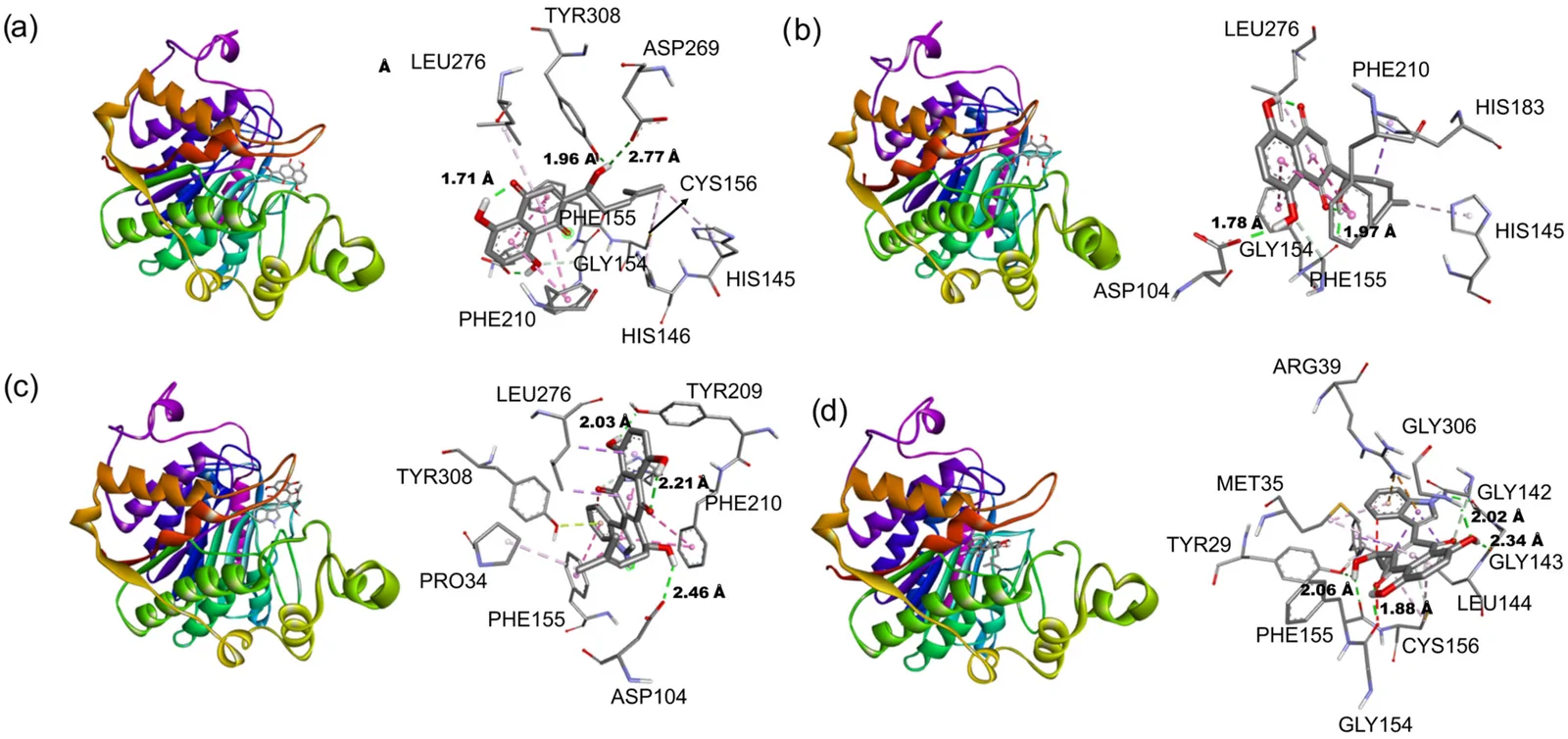
Molecular coupling binding modes and principal interactions with HDAC2 of (**a**) **1**, (**b**) **2,** (**c**) **3,** and (**d**) **4**.

Among the studied compounds, **1** exhibited the highest predicted binding affinity toward HDAC2 (ΔG = −7.46 kcal/mol), followed by **2** (ΔG = −5.31 kcal/mol), **3** (ΔG = −2.59 kcal/mol), and **4** (ΔG = −1.51 kcal/mol). The indole derivatives (**3** and **4**) displayed less favorable binding energies than the parent naphthoquinones (**1** and **2**), suggesting that the incorporation of the indole moiety may reduce the accommodation of these derivatives within the HDAC2 binding pocket. Furthermore, compound **1** (the *S* enantiomer) exhibited a substantially higher predicted binding affinity than compound **2**. The principal ligand–protein interactions are shown in Figure 18 and summarized in Table S10 (Supplementary Materials).

Compound **1** showed several key interactions with BIZ within the lipophilic channel of HDAC2, primarily through hydrophobic contacts with Gly154, Phe155, and Phe210. In contrast, enantiomer **2** interacted with the same three amino acid residues; however, Gly154 formed a hydrogen bond (1.97 Å) with the hydroxyl group of **2**, an interaction that was not observed for **1**. Compound **3** exhibited only favorable π–π interactions with Phe155 and Phe210 within the lipophilic channel. In contrast, **4** formed a conventional hydrogen bond with Phe155 (2.06 Å), as well as distinctive alkyl and π–alkyl interactions with Met35, a residue located in the foot pocket, which were not observed for any of the other studied compounds.

Despite their lower chemical hardness and smaller ΔE_LUMO–HOMO_, compounds **3** and **4** exhibited weaker binding toward HDAC2 than the parent naphthoquinones. These findings indicate that the higher electronic reactivity of the indole derivatives could not compensate for their poorer accommodation within the HDAC2 binding pocket, where steric effects and ligand geometry governed affinity.

## 3. Materials and Methods

### 3.1. Chemistry Quantum

A bibliographic review of the molecules, proteins, and theoretical methods used in this study was carried out. The molecular modeling of each structure was accomplished in Spartan06 [90], and a conformational analysis was performed with the semi-empirical method (AM1/PM3) [91]. As a result, five conformers were selected, based on their lowest energies and differences in spatial distribution. The chosen conformers were optimized using density functional theory with the specialized Gaussian16 software [92] through the Miztli-DGTIC supercomputer. For DFT calculations, Becke’s three-parameter hybrid functional, B3LYP, which includes a mixture of Hartree–Fock exchange and DFT exchange-correlation [93,94], was used. The 6-311++G(d,p) split-valence basis set [95] was employed for all calculations. This basis set includes polarization functions (d and p) and diffuse functions (s and p), which are essential for accurately describing the electronic structure of molecules, particularly those containing highly polarized bonds or anionic species.

In this work, the most relevant geometric, electronic, reactivity, and spectroscopic properties of compounds **1**–**4** were calculated. Among the electronic reactivity descriptors, the electronic chemical potential (μ), ionization potential (IP), electron affinity (EA), electronegativity (χ) [43,51,53], and the energies of the highest occupied molecular orbital (HOMO) and the lowest unoccupied molecular orbital (LUMO) were determined [96]. Furthermore, the electronic chemical potential (μ) and chemical hardness (η) [44,45,46,51] were used to calculate the electrophilicity index (ω) [48]. These descriptors related to molecular stability and reactivity were estimated from the B3LYP/6-311++G(d,p) energies of the neutral, cationic, and anionic species using the corresponding theoretical expressions [43,48].

IP = E_cation_ − E_neutral_ (1)

EA = E_neutral_ − E_anion_ (2)

η = (IP − AE)/2(3)

µ = −(IP + AE)/2(4)

Χ = (IP + AE)/2(5)

ω = (µ^2^/2 η) (6)

Natural population analysis (NPA) is based on natural atomic orbitals (NAOs) derived from the molecular wave function, allowing the determination of atomic charges and orbital populations that depend on the chemical environment of each atom [97]. One of the most widely used properties derived from quantum chemical calculations is the molecular electrostatic potential (MEP). MEP analysis has been extensively employed to identify reactive sites, predict regions susceptible to electrophilic or nucleophilic attack, and investigate molecular recognition processes in biological systems. In MEP maps, red regions correspond to areas of highly negative electrostatic potential, whereas blue regions represent highly positive potential values; intermediate colors, such as orange, yellow, and green, indicate intermediate potential values [98].

Spectroscopic properties, including infrared (IR) and nuclear magnetic resonance (NMR) parameters in both gas and solvent (chloroform) phases, were calculated. Harmonic frequencies, with a scaling factor (0.9679) of the selected modes, were compared with the available experimental information, and the infrared vibrational spectra were generated using GaussSum software version 3.0 [99]. Finally, hydrogen bond interactions were analyzed through wave function calculations at the B3LYP/6-311++G(d,p) level using AIM-2000 software version 2.37 [100].

### 3.2. Chemoinformatic and Biological Activity Prediction

Biological activity prediction was performed using online chemoinformatic tools, including SWISSADME [67], Protox3.0 [68], and Osiris Property Explorer [69]. These platforms were used to estimate physicochemical properties such as molecular weight, log P, hydrogen bond donors and acceptors, and topological polar surface area (TPSA), which were evaluated according to the Lipinski rule of five. PASSOnline [66] was employed to predict the potential biological activities of the studied compounds.

To elucidate the binding mode of compounds **1**–**4**, molecular docking studies were performed using olaparib and naproxen as reference drugs. The three-dimensional structures of the studied compounds were fully optimized using the Gaussian16 program [92] at the B3LYP/6-311++G(d,p) level of theory [93,94]. The crystal structures of the PARP-1 catalytic domain, COX-2, and HDAC2 were retrieved from the Protein Data Bank (PDB IDs: 1UK0, 3NT1, and 3MAX, respectively), and all docking calculations were performed using a single protein monomer.

Molecular docking studies were performed using AutoDock Tools 1.5.7 software due to its reported correlation with experimental binding data. AutoDock Tools 1.5.7, included in the MGLTools package [101], was used to prepare the pdbqt files of the protein structures and ligands for docking calculations. Both the protein and the ligand were treated as rigid during the docking procedure. A directed docking was performed employing a quadratic lattice with points separated by 0.375 Å that was centered on the active site of the enzymes, and the grid center was designated at dimensions (x, y, and z): 5.092, −6.651, and 31.313 Å for PARP-1; −40.628, −49.725, and −23.152 Å for COX-2; and 65.887, 26.645, and 0.214 Å for HDAC2. The binding poses with the lowest binding energy (ΔG) were analyzed employing AutoDock Tools 1.5.7, and the images were created using BIOVIA Discovery Studio Visualizer 2019 [102].

## 4. Conclusions

This study demonstrates the value of combining quantum chemical calculations, chemoinformatics, and molecular docking to investigate the structural, electronic, spectroscopic, and biological properties of alkannin, shikonin, and their corresponding indole derivatives. Density Functional Theory calculations at the B3LYP/6-311++G(d,p) level provided a detailed description of the electronic structure and reactivity of the studied compounds. The molecular electrostatic potential maps, frontier molecular orbitals, and global reactivity descriptors enabled the identification of potential reactive regions involved in protein recognition. Moreover, the calculated NMR chemical shifts showed better agreement with experimental data in chloroform than in the gas phase, confirming the importance of solvent effects for accurately reproducing spectroscopic properties. The AIM analysis further revealed that negative Laplacian values were associated with stronger intramolecular hydrogen bonds, indicating partial covalent character and enhanced electronic delocalization that contribute to molecular stabilization.

The molecular docking studies demonstrated that structural modifications produced distinct effects on protein recognition depending on both the target and the stereochemical configuration of the ligand. Compounds **1** and **3** exhibited the strongest predicted binding affinities toward PARP-1, exceeding that of the reference inhibitor olaparib and establishing key interactions with catalytic residues, particularly His201 and Tyr235. These findings suggest that stereochemistry may play an important role in PARP-1 recognition. All four compounds also displayed favorable binding affinities toward COX-2, outperforming naproxen in the predicted binding energies. Interestingly, incorporation of the indole moiety produced opposite effects on the two parent naphthoquinones, decreasing the affinity of alkannin while enhancing that of shikonin. In contrast, the studied compounds exhibited lower predicted affinities for HDAC2 than the co-crystallized ligand, although several conserved interactions within the lipophilic channel and the foot pocket were identified. Overall, the present results indicate that indole substitution and stereochemical configuration strongly influence the electronic properties and protein-binding behavior of these naphthoquinones. These findings provide valuable insights into the rational design and structural optimization of multitarget anticancer agents based on the naphthoquinone scaffold, which could support future experimental evaluation of the most promising derivatives.

## Figures and Tables

**Table 1:** Energy of the boundary orbitals and ΔE_LUMO–HOMO_ of the studied molecules.

Molecule	HOMO (Hartree)	LUMO (Hartree)	ΔGAP (eV)
**1**	−0.2445	−0.1423	2.779
**2**	−0.2448	−0.1423	2.788
**3**	−0.2217	−0.1265	2.591
**4**	−0.2216	−0.1265	2.589

**Table 2:** Chemical parameters as indicators of reactivity behavior for molecules **1**–**4**.

Molecule	E_neutral_	E_cationic_ ^1^	E_anionic_ ^1^	IP (eV) ^2^	EA (eV) ^3^	η (eV) ^4^	µ (eV) ^5^	Χ (eV) ^6^	ω (eV) ^7^
**1**	−995.74	−995.44	−995.79	8.130	1.480	3.320	−4.809	4.809	3.483
**2**	−995.74	−995.44	−995.79	8.131	1.481	3.319	−4.810	4.808	3.490
**3**	−1358.45	−1358.18	−1358.53	7.380	2.191	2.594	−4.786	4.786	4.414
**4**	−1358.45	−1358.18	−1358.53	7.380	2.191	2.594	−4.786	4.786	4.414

^1^ Theoretical positive and negative energies correspond to the cation and anion states in Hartrees, respectively, at B3LYP/6-311++G(d,p) optimized geometry of the neutral ground state. Therefore, chemical parameters are defined as follows: ^2^ [IP] ionization potential, ^3^ [EA] electron affinity, ^4^ [ƞ] hardness, ^5^ [µ] chemical potential, ^6^ [χ] electronegativity, and ^7^ [ω] electrophilicity index, in eV.

**Table 3:** PASS predictor analysis for target compounds **1**–**4**.

	1,2		3,4	
	Pa	Pi	Pa	Pi
Apoptosis agonist	0.754	0.011	0.654	0.020
Antineoplastic	0.710	0.024	0.605	0.044
Antineoplastic (breast cancer)	0.542	0.015	Not mentioned	Not mentioned
Antineoplastic (lung cancer)	0.462	0.016	Not mentioned	Not mentioned

**Table 4:** Physicochemical and pharmacokinetic most representative properties of compounds **1**–**4**.

	Compound	1	2	3	4
Physicochemical properties	Molecular weight (g/mol)	288.3	288.3	403.3	403.3
	H-acceptors	5	5	5	5
	H-donors	3	3	4	4
	Molar refractivity	77.82	77.82	114.95	114.95
	TPSA (Å)	94.83	94.83	110.62	110.62
	MlogP ^1^/WlogP ^2^	0.42/2.12	0.42/2.12	1.31/4.13	1.31/4.13
Pharmacokinetic properties	Gastrointestinal absorption	High	High	High	High
	Blood–brain barrier permeant	No	No	No	No
	P-gp substrate	No	No	Yes	Yes
	CYP1A2 inhibitor	No	No	No	No
	CYP2C19 inhibitor	No	No	No	No
	CYP2C9 inhibitor	Yes	Yes	No	No
	CYP2D6 inhibitor	No	No	No	No
	CYP3A4 inhibitor	No	No	No	No
	Mutagenic	No	No	No	No
	Tumorigenic	No	No	No	No
	Irritant	No	No	No	No
	Reproductive effect	No	No	No	No
	Drug score	0.583	0.583	0.547	0.547

^1^ MLogP [71] was employed as an atomistic method for analysis and suggested by Lipinski [72]; ^2^ WLogP [73] was suggested for boiled egg diagram representation.

## Data Availability

The data reported in this study are available upon request to adriana.rivera@cuautitlan.unam.mx and nicovain@cuautitlan.unam.mx.

## Supplementary Materials

The following supporting information can be downloaded at https://www.mdpi.com/article/10.3390/molecules31183275/s1, Table S1: Bond lengths (Å) for molecules **1**–**4**; Table S2: Bond angles (°) for molecules **1**–**4**; Table S3: Calculated dihedrals (°) for molecules **1**–**4**; Table S4: Natural atomic charges in e^-^ for molecules **1**–**4**; Table S5: Stretching vibrational frequencies and deviation percentage for the most relevant assignments; Table S6: ^1^H NMR theoretical and experimental chemical shifts (*δ*) for **1**–**4**; Table S7: ^13^C NMR theoretical and experimental chemical shifts (*δ*) for **1**–**4**; Table S8: Electron density, ρ(r), and Laplacian, ∇^2^ρ(r), values at each BCP for compounds **1**–**4**; Table S9: Electron density, ρ(r), and Laplacian, ∇^2^ρ(r), values at each RCP for compounds **1**–**4**. Table S10: Amino acid residues and binding energy values obtained by docking studies between PARP-1, COX-2, and HDAC-2 with target compounds **1**–**4**; Table S11: Cartesian coordinates from compounds **1**–**4**. References [7,34,35,36,54,55,56,80,85,89] are cited in the Supplementary Materials.
