# Supplementary material for: Quantum Theoretical, Chemo-Computational, and Comparative Molecular Coupling Studies of Two Enantiomeric Naphthoquinones, Alkannin and Shikonin, and Their Corresponding Indole Derivatives
Source: Molecules. 2026 Sep 16;31(18):3275. doi: 10.3390/molecules31183275 (PMC13609537; doi:10.3390/molecules31183275)
Supplement: Supplementary file 1 [file molecules-31-03275-s001.zip › molecules-4529940-supplementary.pdf]

Quantum Theoretical, Chemo-Computational, and Comparative Molecular Coupling Studies of Two Enantiomeric Naphthoquinones, Alkannin and Shikonin, and Their Corresponding Indole Derivatives

Adriana Lizbeth Rivera Espejel [1], Sebastian Buendia Lira [1], René Miranda Ruvalcaba [1], René Gerardo Escobedo-González [2], Miguel Cuevas-Cruz [3], Alberto A. Fajardo de la Rosa [4], David A. Nieto-Álvarez [5], Joel Martínez [1,*] and María Inés Nicolás-Vázquez [1,*]

Correspondence: jomtz@comunidad.unam.mx (J.M.); nicovain@cuautitlan.unam.mx (M.I.N.-V.)

CONTENT

Table S1. Bond lengths (Å) for molecules **1-4.**

Table S2. Bond angles (°) for molecules **1-4.**

Table S3. Calculated dihedrals (°) for molecules **1-4.**

Table S4. Natural atomic charges in e^-, for molecules **1-4.**

Table S5. Stretching vibrational frequencies and deviation percentage for the most relevant assignments.

Table S6. ^{1}H NMR theoretical and experimental chemical shifts (δ) for **1-4.**

Table S7. ^{13}C NMR theoretical and experimental chemical shifts (δ) for **1-4.**

Table S8. Electron density, ϱ(r), and Laplacian, ∇^2ϱ(r), values at each BCP for compounds **1-4**.

Table S9. Electron density, ϱ(r), and Laplacian, ∇^2ϱ(r), values at each RCP for compounds **1-4**.

Table S10. Amino acid residues and binding energy values obtained by docking studies between PARP-1, COX-2, and HDAC2 with target compounds **1-4.**

Table S11. Cartesian coordinates from compounds **1-4.**

Table S1. Bond lengths (Å) for molecules **1-4.**

	**1**	**2**		**3**	**4**	
**Bond**	**T (Å)**	**T (Å)**	**Bond**	**T (Å)**	**T (Å)**	**Reference**
C1-C2	1.459	1.459	C1-C2	1.458	1.458	1.448[a]
C2-C3	1.401	1.401	C2-C3	1.404	1.404	1.460[a]
C3-C4	1.417	1.417	C3-C4	1.415	1.415	1.405[a]
C4-C5	1.371	1.371	C4-C5	1.371	1.371	1.318[a]
C5-C6	1.416	1.416	C5-C6	1.415	1.415	1.400[a]
C6-C7	1.405	1.405	C6-C7	1.403	1.403	1.364[a]
C7-C8	1.457	1.457	C7-C8	1.456	1.456	1.492[a]
C8-C9	1.485	1.485	C8-C9	1.472	1.472	1.451[a]
C9-C10	1.347	1.347	C9-C10	1.370	1.370	1.306[a]
C9-C11	1.519	1.519	C10-C11	1.462	1.462	1.448[b]
C11-C12	1.551	1.551	C11-C12	1.382	1.382	1.535[c]
C12-C13	1.502	1.502	C12-N1	1.366	1.366	1.363[b]
C13-C14	1.340	1.339	N1-C13	1.384	1.384	1.370[b]
C14-C15	1.509	1.509	C13-C14	1.394	1.394	1.312[c]
C14-C16	1.507	1.507	C14-C15	1.388	1.388	1.495[c]
C1-O1	1.243	1.243	C15-C16	1.406	1.406	-
C3-O3	1.336	1.336	C16-C17	1.388	1.388	-
C6-O6	1.336	1.336	C17-C18	1.404	1.404	-
C8-O8	1.249	1.249	C18-C11	1.455	1.455	-
C11-O11	1.422	1.422	C9-C19	1.524	1.524	-
O3-H	0.990	0.990	C19-C20	1.553	1.533	-
O6-H	0.988	0.988	C20-C21	1.503	1.503	-
O11-H	0.967	0.967	C21-C22	1.339	1.339	-
			C22-C23	1.509	1.509	-
			C22-C24	1.507	1.507	-
			C1-O1	1.242	1.242	-
			C3-O3	1.337	1.337	-
			C6-O6	1.337	1.337	-
			C8-O8	1.255	1.255	-
			C19-O19	1.427	1.427	-
			O3-H	0.989	0.989	-
			O6-H	0.990	0.990	-
			O19-H	0.967	0.967	-
			N1-H	1.006	1.006	-

[T]: Theoretical. [a] obtained from Vijayalakshmi et al., 1987 [1], [b] obtained from Soriano-García et al., 1986 [2]; [c] obtained from Noltemeyer et al., 1982[3].

Table S2. Bond angle (°) for molecules **1-4.**

	**1**	**2**		**3**	**4**
**Angle**	**T (°)**	**T (°)**	**Bond**	**T (°)**	**T (°)**
C10-C1-O1	120.1	120.1	C10-C1-O1	121.3	121.2
O1-C1-C2	121.8	121.8	O1-C1-C2	121.8	121.8
C2-C3-O3	122.8	122.8	C2-C3-O3	125.8	125.8
O3-C3-C4	117.8	117.8	O3-C3-C4	113.6	113.6
C3-C4-C5	120.7	120.7	C3-C4-C5	119.9	119.8
C4-C5-C6	120.8	120.8	C4-C5-C6	120.0	120.0
C6-C7-C2	119.6	119,6	C6-C7-C2	119.1	119.2
C7-C8-O8	121.1	121.1	C7-C8-O8	121.5	121.4
O8-C8-C9	119.8	119.8	O8-C8-C9	120.6	120.6
C10-C9-C11	121.9	121.9	C9-C10-C11	125.3	125.3
C9-C11-O11	111.5	111.6	C10-C11-C12	125.3	125.3
C11-C12-C13	114.2	113.6	C11-C12-N1	110.2	110.2
C12-C13-C14	127.6	128.7	C12-N1-C13	108.6	108.5
C13-C14-C15	120.7	120.7	C12-C11-C18	106.7	106.7
C13-C14-C16	124.7	124.8	C13-C14-C15	117.6	117.6
			C12-C14-C15	121.4	121.5
			C14-C15-C16	121.4	121.4
			C15-C16-C17	118.9	118.9
			C16-C17-C18	119.1	119.1
			C13-C18-C17	109.7	109.6
			C9-C19-C20	111.5	111.4
			C19-C20-C21	123.4	123.6
			C20-C21-C22	122.2	122.3
			C21-C22-C24	121.7	121.7
			C9-C19-O9	111.3	111.4

[T]: Theoretical

Table S3. Calculated dihedrals (°) for molecules **1-4.**

	**1**	**2**		**3**	**4**
**Angle**	**T (°)**	**T (°)**	**Bond**	**T (°)**	**T (°)**
C1-C2-C3-O3	0.133	-0.192	C1-C2-C3-O3	-0.067	0.131
C1-C2-C3-C4	-179.8	179.8	C1-C2-C3-C4	-179.2	179.3
O1-C1-C2-C3	-0.160	0.239	O1-C1-C2-C3	-22.9	22.7
C2-C3-C4-C5	0.077	-0.076	C2-C3-C4-C5	-1.73	1.710
C3-C4-C5-C6	-0.015	0.029	C3-C4-C5-C6	-0.10	-0.102
C4-C5-C6-O6	179.9	179.9	C4-C5-C6-O6	-178.8	178.8
O6-C6-C7-C2	-179.9	-179.9	O6-C6-C7-C2	178.9	-178.9
O6-C6-C7-C8	-0.041	0.048	O6-C6-C7-C8	-0.764	0.779
C6-C7-C8-O8	-0.327	0.110	C6-C7-C8-O8	16.26	-15.79
O8-C8-C9-C10	-179.1	179.3	O8-C8-C9-C10	167.5	-168.09
O8-C8-C9-C11	1.854	-1.839	C9-C10-C11-C12	132.9	-133.20
C10-C9-C11-O11	129.0	-129.3	C10-C11-C12-N1	177.4	-177.42
C10-C9-C11-C12	-104.6	104.2	C11-C12-N1-C13	-1.08	1.059
C9-C11-C12-C13	60.5	-67.0	C13-C14-C15-C16	0.150	-0.090
O11-C11-C12-C13	-173.0	166.6	C14-C15-C16-C17	0.096	-0.180
C11-C12-C13-C14	102.6	119.2	C16-C17-C18-C11	-178.64	178.6
C12-C13-C14-C15	179.6	179.1	C17-C18-C11-C10	1.897	-1.86
C12-C13-C14-C16	0.036	-0.020			

[T]: Theoretical

Table S4. Natural atomic charges in e^-, for molecules **1-4.**

Atom	1	2	Atom	3	4
C1	0.419	0.418	C1	0.473	0.473
C2	-0.174	-0.179	C2	-0.174	-0.174
C3	0.338	0.338	C3	0.331	0.331
C4	-0.256	-0.255	C4	-0.264	-0.264
C5	-0.260	-0.261	C5	-0.233	-0.233
C6	0.336	0.348	C6	0.326	0.325
C7	-0.175	-0.170	C7	-0.169	-0.169
C8	0.428	0.393	C8	0.441	0.441
C9	-0.113	-0.021	C9	-0.111	-0.111
C10	-0.253	-0.282	C10	-0.124	-0.123
C11	0.133	0.114	C11	-0.116	-0.116
C12	-0.432	-0.432	C12	0.027	0.027
C13	-0.174	-0.184	C13	0.154	0.155
C14	-0.037	-0.021	C14	-0.249	-0.249
C15	-0.584	-0.584	C15	-0.229	-0.229
C16	-0.597	-0.598	C16	-0.237	-0.237
O1	-0.707	-0.707	C17	-0.191	-0.191
O3	-0.711	-0.711	C18	-0.075	-0.075
O6	-0.711	-0.714	C19	0.102	0.102
O8	-0.755	-0.757	C20	-0.441	-0.440
O11	-0.759	-0.750	C21	-0.182	-0.182
H3	0.502	0.502	C22	-0.056	-0.056
H6	0.503	0.506	C23	-0.611	-0.611
H11	0.482	0.482	C24	-0.581	-0.581
			N1	-0.555	-0.555
			N1-H	0.390	0.390
			O1	-0.703	-0.703
			O3	-0.710	-0.718
			O6	-0.713	-0.713
			O8	-0.745	-0.745
			O19	-0.757	-0.757
			H3	0.499	0.499
			H6	0.502	0.502
			H19	0.475	0.475

[1] Theoretical values

Table S5. Stretching vibrational frequencies and deviation percentage for the most relevant assignments for compounds **1-4**.

Molecule	Assignment	Experimental frequency (cm^{-1}) [1]	Theoretical frequency (cm^{-1}) [2]	Deviation (%)
**1**	Aromatic and quinone double bonds	1413-1576	1430-1525	1.2013, -3.2360
	Carbonyl groups	1603-1653	1586-1638	-1.0605, -0.9074
	Vinyl C=C	-	1669	-
	Terminal Csp3-H	-	2910-2917	-
	Aromatic Csp2-H	-	3075-3100	-
	Quinone OH	-	3184-3224	
	Lateral chain OH	-	3639	
**2**	Aromatic and quinone double bonds	1413-1576	1439-1525	1.8400, -3.2360
	Carbonyl groups	1603-1653	1586-1638	-1.0651, -0.9074
	Vinyl C=C	-	1669	-
	Terminal Csp3-H	-	2909-2916	-
	Aromatic Csp2-H	-	3075-3100	-
	Quinone OH	-	3184-3224	-
	Lateral chain OH	-	3636	-
**3**	Indole aromatic Csp2-H	-	1601-1559	-
	Aromatic and quinone double bonds	-	1437-1523	-
	Carbonyl groups	-	1584-1636	-
	Vinyl C=C	-	1671	-
	Quinone OH	-	3192-3187	-
	NH	-	3547	-
	Lateral chain OH	-	3626	-
**4**	Indole aromatic Csp2-H	-	1600-1558	-
	Aromatic and quinone double bonds	-	1437-1523	-
	Carbonyl groups	-	1584-1636	-
	Vinyl C=C	-	1670	-
	Quinone OH	-	3191-3186	-
	NH	-	3547	-
	Lateral chain OH	-	3626	-

[1] From References [4]. [2] Corrected theoretical vibrational frequencies.

Table S6. ^{1}H NMR theoretical (gas phase/solvent) and experimental chemical shift (δ) for **1-4**.

	**1**		**2**			**3**	**4**
			**δ (ppm)**				
**Assignment**	**E [1]**	**T [2]**	**E [3,4]**	**T [2]**	**Assignment**	**T [2]**	**T [2]**
C16-H	1.65	1.7/2.4	1.66	1.7/2.4	C23-H	1.0/1.3	1.0/1.3
C16-H′	1.65	1.7/1.6	1.66	1.7/1.7	C23-H′	1.3/1.7	1.3/1.7
C16-H′′	1.65	1.8/1.6	1.66	1.8/1.6	C24-H	1.4/1.1	1.4/1.0
C15-H	1.75	1.1/1.7	1.76	1.0/1.7	C24-H′	1.6/1.6	1.6/1.6
C15-H′	1.75	1.3/1.8	1.76	1.3/1.8	C24-H′	1.7/1.7	1.7/1.7
C15-H′′	1.75	1.9/1.9	1.76	1.9/1.9	C23-H′′	2.0/1.9	2.0/1.9
C12-H12	2.3	2.6/2.6	2.42	2.6/2.6	C20-H	2.4/2.3	2.4/2.3
C12-H12′	2.7	3.1/3.0	2.47	3.1/2.9	C20-H′	3.2/2.7	3.2/2.8
C11-H11	4.9	4.1/4.1	5.11	5.4/5.1	O19-H	4.1/4.0	4.1/4.0
C13-H13	5.2	5.4/5.1	6.08	4.1/5.1	C19-H19	4.9/4.7	4.9/4.7
C10-H10	7.1	6.5/6.8	6.98	6.5/6.8	C21-H21	5.0/5.9	5.0/5.7
C5-H15	7.2	7.3/7.6	7.19	7.3/7.6	C5-H5	7.4/7.6	7.4/7.6
C4-H4	7.2	7.4/7.6	6.08	7.4/7.6	C4-H4	7.4/7.6	7.4/7.6
O6-H	-	12.9/12.8	12.49	12.7/12.8	C14-H14	7.5/7.7	7.5/7.7
O3-H	-	12.7/12.8	12.60	12.8/12.8	C16-H16	7.5/7.6	7.5/7.6
					C15-H15	7.6/7.6	7.6/7.6
					N-H	7.8/8.7	7.8/8.6
					C12-H12	7.8/7.9	7.8/7.9
					C17-H17	8.6/8.2	8.6/8.2
					O6-H	13.0/13.1	13.0/13.0
					O3-H	13.0/13.1	13.0/13.0

[1] Experimental. From Reference [5]. [2] Theoretical. [3,4] From Reference [6-7].

Table S7. ^{13}C NMR theoretical (gas phase/solvent) and experimental chemical shift (δ) for **1-4**.

	**1**		**2**			**3**	**4**
			**δ (ppm)**				
**Assignment**	**E** [1]	**T** [2]	**E** [3]	**T** [2]	**Assignment**	**T** [2]	**T** [2]
C1	180.5	194.7/195.6	179.8	194.7/195.6	C1	194.2	194.2
C2	112.0	117.1/118.0	111.6	117.1/118.0	C2	117.6	117.6
C3	164.9	165.9/166.8	165.6	165.9/166.8	C3	166.4	166.4
C4	132.28	137.6/138.5	132.4	137.6/138.5	C4	135.8	135.8
C5	131.85	135.7/136.6	132.3	135.7/136.6	C5	136.3	136.3
C6	165.5	167.0/167.9	164.9	167.0/167.9	C6	165.7	165.7
C7	111.5	118.5/119.4	112.0	118.5/119.4	C7	118.4	118.4
C8	179.7	197.5/198.4	180.6	197.5/198.4	C8	197.4	197.4
C9	151.4	159.6/160.4	151.4	159.5/160.4	C9	151.6	151.6
C10	132.35	142.9/143.8	131.9	143.0/143.9	C10	149.1	149.1
C11	68.3	83.9/84.8	68.4	84.0/84.8	C11	115.8	115.8
C12	35.6	42.9/43.8	35.7	42.9/43.8	C12	138.9	138.9
C13	118.5	128.2/129.1	118.5	128.3/129.1	C13	142.6	142.6
C14	137.5	147.2/148.0	137.4	147.2/148.1	C14	114.9	114.9
C15	18.1	19.4/20.2	18.1	19.3/20.2	C15	129.5	129.5
C16	25.9	28.8/29.7	25.9	28.8/29.7	C16	128.9	128.9
					C17	128.6	128.6
					C18	133.7	133.7
					C19	79.0	79.0
					C20	43.1	43.1
					C21	129.5	129.5
					C22	144.4	144.4
					C23	19.2	19.2
					C24	28.8	28.8

[1] Experimental. From Reference [5]. [2] Theoretical. [3] From Reference [6].

Table S8. Electron density, ϱ(r), and Laplacian, $\nabla^2\varrho(r)$, values at each BCP for compounds **1-4**.

**Compound**	**BCP**	**ϱ(r) (e.a.u.$^{-3}$)**	**$\nabla^2\varrho(r)$ (e.a.u.$^{-5}$)**
	1a	0.0484	−0.0348
**1**	1b	0.0484	−0.0348
	1c	0.0213	−0.0190
	2a	0.0482	−0.0347
**2**	2b	0.0484	−0.0356
	2c	0.0214	−0.0191
	3a	0.0507	−0.0360
**3**	3b	0.0510	−0.0360
	3c	0.0230	−0.0210
	4a	0.0501	−0.0360
**4**	4b	0.0510	−0.0360
	4c	0.0230	−0.0210

Table S9. Electron density, ϱ(r), and Laplacian, $\nabla^2\varrho(r)$, values at each RCP for compounds **1-4**.

**Compound**	**RCP**	**ϱ(r) (e.a.u.$^{-3}$)**	**$\nabla^2\varrho(r)$ (e.a.u.$^{-5}$)**
	1a	0.0192	−0.0294
**1**	1b	0.0191	−0.0293
	1c	0.0142	−0.0190
	2a	0.0192	−0.0293
**2**	2b	0.0291	−0.0293
	2c	0.0142	−0.0191
	3a	0.0190	−0.0290
**3**	3b	0.0190	−0.0301
	3c	0.0150	−0.0201
	4a	0.0190	−0.0290
**4**	4b	0.0190	−0.0301
	4c	0.0150	−0.0201

Table S10. Amino acid residues and binding energy values obtained by docking studies between PARP-1, COX-2, and HDAC2 with target compounds **1-4.**

**Molecule**	**Binding energy (kcal/mol) with PARP-1**	**Amino acids' interaction with PARP-1** [1]	**Binding energy (kcal/mol) with COX-2**	**Amino acids' interaction with COX-2** [1]	**Binding energy (kcal/mol) with HDAC2**	**Amino acids' interaction with HDAC2** [1]
**1**	-11.44	**His201**, Gly202, Arg204, Tyr246, Ala237	-11.2	**Val523**, Val349, Leu352, **Tyr355**, Met522, Ser530, His90	-7.46	**Phe210, Gly154, Phe155**, His145, Cys156, His146
**2**	-7.94	**His201**, **Tyr235**, Tyr246, Gly202, Tyr228	-8.44	**Val523**, Val349, Leu352, **Tyr355**, Met522, Ser530, Phe530, Tyr385	-5.31	**Phe210, Gly154, Phe155**, His145, Leu276
**3**	-10.85	**His201**, **Tyr235**, Tyr246, Gly202, **Glu327**, Ala237, Ile234	-9.64	**Val523**, Val349, Leu352, Met522, Ser530, Trp387, Ser353, Leu531	-2.59	**Phe210, Phe155**, Leu276, Tyr308, Tyr209, Asp104
**4**	-10.54	**His201**, Tyr246, Gly202, Tyr228, Ala237, Asp105	-10.14	**Val523**, Val349, Leu352, **Tyr355**, Ser353, Val527	-1.51	**Phe155**, Cys156, **Met35, Leu144, Gly154**, Gly142, Gly143
**Olaparib**	-10.23	**His201**, **Tyr235**, Tyr246, Gly202, Ile 234, Asp105	-	-	-	-
**Naproxen**	-	-	-8.75	**Val523**, Leu531, Trp387, Val349, Leu352, **Tyr355**, **Arg120**	-	-
**KIZ**	-	-	-	-	-8.07	**Phe210**, **Gly154**, **Phe155**, His145, **Leu144**, Cys156, **Met35**

[1] Compared from Reference [8-10]. Main amino acid residues in the pocket site are shown in bold.

Table S11. Cartesian coordinates of optimized compounds **1-4.**

Compound **1**

C 2.31267500 -1.42791900 0.88966600
C 3.08390600 1.14968000 0.03407300
C 1.44483300 -0.66907200 0.08578900
C 3.56704900 -0.88463800 1.26092400
C 3.94246300 0.36724400 0.84598500
C 1.83825600 0.63110300 -0.34339100
C 0.15191800 -1.21408400 -0.30724400
C 0.94319900 1.42073200 -1.18410100
O -0.20639700 -2.35419700 0.05768900
O 1.24892600 2.55879200 -1.58012100
C -0.34840900 0.83026900 -1.55865500
C -0.74684500 -0.39325400 -1.15853500
O 3.50821500 2.36049300 -0.33880300
C -2.10692900 -0.95875500 -1.53021600
C -3.10625700 -0.93065100 -0.34406100
C -3.51146200 0.45614100 0.06925500
C -3.34321400 1.05681000 1.25521200
C -2.67688800 0.42905400 2.45310500
C -3.84340200 2.46164900 1.48644500
H 4.21819300 -1.48884400 1.88096600
H 4.90117600 0.78692300 1.12602000
H 2.79302700 2.75922700 -0.89605100
H -0.97824100 1.45174100 -2.18661500
H -3.98724100 -1.47604200 -0.70270900
H -2.69427600 -1.50747600 0.48528400
H -4.01790100 1.02829800 -0.70801700
H -4.31566300 2.87767000 0.59450200
H -3.02329000 3.12629300 1.78207200
H -4.57478200 2.48568900 2.30283900
H -2.31298500 -0.58136700 2.26950300
H -1.82904100 1.04080300 2.78263800
H -3.37362800 0.38635000 3.29829500
O -1.99536800 -2.26063300 -2.09245400
H -1.58340000 -2.82441700 -1.42312400
H -2.51384100 -0.33672500 -2.33182800
O 2.01554900 -2.65374100 1.33103300
H 1.11912100 -2.87968800 0.98198300

Compound **2**

C -2.31260200 -1.42800800 0.88968200
C -3.08402900 1.14959900 0.03425800
C -1.44484500 -0.66904700 0.08580000
C -3.56701100 -0.88481400 1.26100700
C -3.94250900 0.36706700 0.84615600
C -1.83837300 0.63111700 -0.34329400
C -0.15191800 -1.21398700 -0.30731200
C -0.94341500 1.42082700 -1.18403300
O 0.20645100 -2.35409800 0.05753000
O -1.24921400 2.55889000 -1.57997900
C 0.34822500 0.83046700 -1.55865700
C 0.74676000 -0.39304700 -1.15862000
O -3.50840300 2.36042400 -0.33851300
C 2.10684700 -0.95845800 -1.53044100
C 3.10622800 -0.93054600 -0.34434000
C 3.51145600 0.45618800 0.06916600
C 3.34346200 1.05660200 1.25528700
C 2.67735900 0.42861000 2.45317900
C 3.84366400 2.46140700 1.48670000
H -4.21809200 -1.48910700 1.88103400
H -4.90124300 0.78664800 1.12626400
H -2.79323700 2.75923400 -0.89573200
H 0.97798500 1.45201500 -2.18661700
H 2.51371500 -0.33629000 -2.33196800
O 1.99526500 -2.26024000 -2.09290800
H 1.58343900 -2.82417300 -1.42361800
H 2.69427500 -1.50748600 0.48493900
H 3.98719800 -1.47588400 -0.70310400
H 4.01769500 1.02852600 -0.70810300
H 4.31553100 2.87770600 0.59467900
H 4.57538000 2.48526000 2.30279800
H 3.02363800 3.12592200 1.78285600
H 1.82923000 1.04000800 2.78264600
H 2.31389900 -0.58197400 2.26960000
H 3.37407800 0.38625200 3.29840000
O -2.01538800 -2.65382800 1.33097200
H -1.11897100 -2.87969200 0.98187900

Compound **3**

C -3.76134100 -0.53029600 -1.23825800
C -2.99273900 -2.35047900 0.77656100
C -2.44586500 -0.52096400 -0.74885900
C -4.68773200 -1.46177300 -0.71086600
C -4.31420100 -2.34606200 0.26889800
C -2.05621900 -1.43961100 0.26066900
C -1.47841100 0.44427900 -1.25335800
C -0.68023700 -1.45278100 0.74553100
O -1.81550900 1.26379500 -2.14216100

O -0.34147900 -2.24075000 1.64439500
C 0.32155600 -0.50448300 0.15093100
C -0.10317000 0.45928900 -0.72609400
O -2.69913900 -3.23925100 1.73182900
C 0.77247100 1.62081400 -1.18319900
C 0.30124900 2.97606600 -0.58865900
C 0.46157300 3.06370400 0.90334800
C -0.48278700 3.26147100 1.83297800
C -1.95264500 3.43915600 1.54751200
C -0.12004000 3.33838200 3.29628000
H -5.69773800 -1.45191100 -1.10263400
H -5.01745600 -3.06234400 0.67631400
H -1.74495300 -3.10441600 1.95841300
H 0.93926500 3.72921500 -1.06657000
H -0.72071300 3.17981500 -0.90771700
H 1.48723400 2.95551800 1.25503500
H -0.40718100 4.30807600 3.72011500
H 0.95119200 3.20107300 3.45641600
H -0.65487700 2.57539100 3.87458900
H -2.54143000 2.68733200 2.08610100
H -2.29647000 4.41634700 1.90677900
H -2.20137800 3.36662500 0.48932900
O 0.87315600 1.68332000 -2.60542300
H -0.02978400 1.78491800 -2.93852200
H 1.78772800 1.45153100 -0.83404400
C 2.89946500 -0.74532000 -0.28000200
C 5.50076100 -1.06694100 -1.27039700
C 3.99394300 -1.04610800 0.56892100
C 3.13593000 -0.62044700 -1.65899700
C 4.42968100 -0.78172100 -2.13597300
C 5.29620800 -1.20527100 0.09591900
C 1.71269900 -0.69669000 0.56077800
C 2.14166800 -0.97942300 1.84424800
N 3.49585400 -1.16272700 1.85584000
H 4.04051400 -1.37233600 2.67610700
H 2.33156700 -0.38248800 -2.34424200
H 4.61946800 -0.68049100 -3.19826200
H 6.49940300 -1.18350600 -1.67518900
H 6.11633100 -1.43694000 0.76656300
H 1.56768600 -1.05577000 2.75140800
O -4.19505900 0.30813800 -2.18607100
H -3.42604800 0.88269100 -2.42887600

Compound **4**

C 3.76148400 -0.53023800 -1.23781700
C 2.99254900 -2.35080600 0.77649000
C 2.44595000 -0.52097300 -0.74863400
C 4.68779900 -1.46177500 -0.71047900
C 4.31411000 -2.34627400 0.26904200
C 2.05611600 -1.43982700 0.26064500
C 1.47857700 0.44435400 -1.25309500
C 0.68006800 -1.45307300 0.74531500
O 1.81586200 1.26405600 -2.14166400
O 0.34117100 -2.24126700 1.64391900
C -0.32155500 -0.50449600 0.15089800
C 0.10324700 0.45931300 -0.72603000
O 2.69884000 -3.23977000 1.73155100
C -0.77233900 1.62081900 -1.18324000
C -0.30106300 2.97612500 -0.58890400
C -0.46157900 3.06398900 0.90306800
C 0.48268800 3.26177000 1.83278800
C 1.95261500 3.43916900 1.54749700
C 0.11977000 3.33890300 3.29603600
H 5.69786500 -1.45183200 -1.10208700
H 5.01724900 -3.06266100 0.67647000
H 1.74464100 -3.10496500 1.95811300
H -1.78761200 1.45160400 -0.83410800
O -0.87302300 1.68307400 -2.60550300
H 0.02991100 1.78474400 -2.93859600
H 0.72097100 3.17969200 -0.90784300
H -0.93891700 3.72928200 -1.06701400
H -1.48729700 2.95600300 1.25464500
H -0.95154100 3.20205400 3.45603100
H 0.40728100 4.30847700 3.71988300
H 0.65419600 2.57569400 3.87444500
H 2.29657100 4.41631700 1.90675000
H 2.54117800 2.68726200 2.08621500
H 2.20147900 3.36651200 0.48935400
C -2.89946600 -0.74534500 -0.28005300
C -1.71273400 -0.69660900 0.56074800
C -2.14171900 -0.97913500 1.84423500
N -3.49592600 -1.16240600 1.85583300
C -3.99397400 -1.04597900 0.56889200
C -3.13587500 -0.62064700 -1.65907400
C -4.42961500 -0.78193300 -2.13606900
C -5.50073200 -1.06699300 -1.27048000
C -5.29623200 -1.20515100 0.09585900
H -4.61937100 -0.68084700 -3.19837700
H -2.33146300 -0.38284900 -2.34432200
H -6.49936600 -1.18357200 -1.67528700
H -6.11639200 -1.43669400 0.76650300
H -4.04060700 -1.37185500 2.67612700
H -1.56776100 -1.05539300 2.75142400
O 4.19535200 0.30838600 -2.18539400
H 3.42630500 0.88291200 -2.42827200

References

1. Vijayalakshmi, J.; Rajan, S.S.; Srinivasan, R. Structure of plumbagin. Acta Crystallogr. C 1987, 43, 2375–2377.
2. Soriano-García, M.; Toscano, R.A.; Flores-Valverde, E.; Montoya-Vega, F.; López-Celis, I.; IUCr Structure of 2-(1,5-dimethyl-4-hexenyl)-3-hydroxy-5-methyl-1,4-benzoquinone (perezone), a sesquiterpene. *Acta Cryst.* **1986**, *42*, 327–329, doi:10.1107/S0108270186096300.
3. Noltemeyer, M.; Sheldrick, G.; Hoppe, H.-U.; Zeeck, A. 2-Ethyl-5-(3-indolyl)oxazole from *Streptomyces cinnamomeus* discovered by chemical screening. *J. Antibiot.* (Tokyo). **1982**, *35*, 549–555.
4. Cañamares, M.V.; Mieites-Alonso, M.G.; Leona, M. Raman, SERS and DFT analysis of the natural red dyes of Japanese origin alkannin and shikonin. *Spectrochim. Acta Part A Mol. Biomol. Spectrosc.* **2022**, *265*, 120382-120382. https://doi.org/10.1016/j.saa.2021.120382.
5. Spyros, A.; Assimopoulou, A.N.; Papageorgiou, V.P. Structure determination of oligomeric alkannin and shikonin derivatives. *Biomed. Chromatogr.* **2005**, *19* (7). 498–505. https://doi.org/10.1002/bmc.470.
6. Cho, M.-H.; Paik, Y.-S.; Hahn, T.-R. Physical stability of shikonin derivatives from the roots of *Lithospermum erythrorhizon* cultivated in Korea. *J. Agric. Food Chem.* **1999**, *47* (10), 4117–4120. https://doi.org/10.1021/jf9902853.
7. Pietrosiuk, A.; Wiedenfeld, H. Shikonin derivatives from *Lithospermum canescens*. *Pharm. Biol.* **2005**, *43* (2), 189–191. https://doi.org/10.1080/13880200590919555.
8. Ahmadi, M.; Bekeschus, S.; Weltmann, K.-D.; von Woedtke, T.; Wende, K. Non-steroidal anti-inflammatory drugs: recent advances in the use of synthetic COX-2 inhibitors. *RSC Med. Chem.* **2022**, *13*, 471–496. https://doi.org/10.1039/d1md00280e.
9. Rubiales-Martínez, A.; Martínez, J.; Mera-Jiménez, E.; Pérez-Flores, J.; Téllez-Isaías, G.; Miranda Ruvalcaba, R.; Hernández-Rodríguez, M.; Mancilla Percino, T.; Macías Pérez, M.E.; Nicolás-Vázquez, M.I. Design of two new sulfur derivatives of perezone: *In silico* study simulation targeting PARP-1 and in vitro study validation using cancer cell lines. *Int. J. Mol. Sci.* **2024**, *25* (2), 868. https://doi.org/10.3390/ijms25020868.
10. Bressi, J.C.; Jennings, A.; Skene, R.J.; Wu, Y.; Melkus, R.; Ron de Jong; O'Connell, S.M.; Grimshaw, C.E.; Navre, M.; Gangloff, A.R. Exploration of the HDAC2 foot pocket: synthesis and SAR of substituted *N*-(2-aminophenyl)benzamides. *Bioorg. Med. Chem. Lett.* **2010**, *20* (10), 3142–3145. https://doi.org/10.1016/j.bmcl.2010.03.091.
